# Methods to measure blood flow and vascular reactivity in the retina

**DOI:** 10.3389/fmed.2022.1069449

**Published:** 2023-01-12

**Authors:** Elsa Wilma Böhm, Norbert Pfeiffer, Felix Mathias Wagner, Adrian Gericke

**Affiliations:** Department of Ophthalmology, University Medical Center, Johannes Gutenberg University of Mainz, Mainz, Germany

**Keywords:** blood flow, measurement, perfusion, retina, vasculature, vessel diameter

## Abstract

Disturbances of retinal perfusion are involved in the onset and maintenance of several ocular diseases, including diabetic retinopathy, glaucoma, and retinal vascular occlusion. Hence, knowledge on ocular vascular anatomy and function is highly relevant for basic research studies and for clinical judgment and treatment. The retinal vasculature is composed of the superficial, intermediate, and deep vascular layer. Detection of changes in blood flow and vascular diameter especially in smaller vessels is essential to understand and to analyze vascular diseases. Several methods to evaluate blood flow regulation in the retina have been described so far, but no gold standard has been established. For highly reliable assessment of retinal blood flow, exact determination of vessel diameter is necessary. Several measurement methods have already been reported in humans. But for further analysis of retinal vascular diseases, studies in laboratory animals, including genetically modified mice, are important. As for mice, the small vessel size is challenging requiring devices with high optic resolution. In this review, we recapitulate different methods for retinal blood flow and vessel diameter measurement. Moreover, studies in humans and in experimental animals are described.

## 1. Introduction

### 1.1. Impaired blood flow in ocular diseases

Disturbances of retinal perfusion have been implicated in the pathophysiology of various ocular diseases. Hence, assessment of perfusion abnormalities is important to monitor disease progression and treatment success. Although measurement of retinal perfusion has been very challenging for many years, recently new methods have been developed that will enhance our understanding on the pathophysiology of retinal diseases. In this article, we present different methods that are used to measure retinal vascular reactivity and blood flow. Moreover, we discuss advantages and weaknesses of individual methods. The following retinal diseases have been associated with abnormalities of retinal perfusion and are often investigated by using blood flow and/or vascular reactivity measurement techniques.

#### 1.1.1. Diabetic retinopathy

Diabetic retinopathy is a widespread complication of diabetes mellitus and one of the primary causes of visual loss worldwide, which is characterized by morphological and functional changes of small blood vessels (1). Chronic hyperglycemia induces oxidative stress reducing function and viability of vascular and neuronal cells. A loss of pericytes pre-disposes to microaneurysm formation. Moreover, endothelial cell loss, basement membrane thickening, capillary occlusion and consecutive ischemia may occur (1, 2). Ischemic conditions trigger generation of proangiogenic substances such as vascular endothelial growth factor (VEGF), resulting in neovascularization, which initiates the proliferative stage of diabetic retinopathy. Moreover, damage of the blood-retina barrier (BRB) is the main cause of diabetic macular edema (3). Because of coexisting endothelial dysfunction, vascular regulatory actions such as functional hyperemia and autoregulation of vascular tone are impaired (4–6).

#### 1.1.2. Retinal vein occlusion

Retinal vein occlusion (RVO) is a common cause of vision loss and is associated with increasing age and cardiovascular risk factors (7). There are two types of thrombotic vein occlusion: central retinal vein occlusion (CRVO) or branch retinal vein occlusion (BRVO) (7). For pathophysiologic mechanisms, systemic vascular diseases with consequent atherosclerosis and damage of endothelial cells (8) and a prothrombotic state (9) are discussed. The clinical presentation is characterized by a dilatation and tortuosity of retinal veins, retinal and subretinal hemorrhage and macular edema (10). Classification in ischemic and non-ischemic disease stages is important. Due to increasing vascular resistance during retinal vein occlusion, retinal perfusion decreases, and occlusion of capillaries occurs. This may also lead to vascular remodeling with acute endothelial cell apoptosis, loss of pericytes and increased vascular permeability resulting in edema or ischemia (11). By consequent angiogenic stimuli through VEGF, complications such as formation of neovascularization can occur (7).

#### 1.1.3. Retinal artery occlusion

Acute retinal arterial ischemia includes transient monocular vision loss (TMVL), branch retinal arterial occlusion (BRAO), and central retinal arterial occlusion (CRAO) (12). Due to an association with a higher risk of stroke and cardiac events, acute disease management and assessment of risk factors is important in affected patients (7, 12). A major cause of retinal artery occlusion are emboli from the heart, aortic arch or carotid artery, but also systemic vasculitis (7). Sudden interruption of retinal blood flow, often results in permanent retinal ischemia with consequent irreversible cell death (7).

#### 1.1.4. Glaucoma

Glaucoma is another frequent cause of blindness associated with vascular regulatory dysfunction. Elevated intraocular pressure (IOP) is the main risk factor for retinal ganglion cell ganglion cell and consecutive visual field loss. Strikingly, our group recently demonstrated that even moderately increased IOP induced endothelial dysfunction and abnormal autoregulation in retinal arterioles, suggesting that IOP and retinal perfusion are in strong interaction (13). Intriguingly, even one month after normalization of initially elevated IOP endothelial dysfunction and abnormal autoregulation persisted in retinal blood vessels, indicative of long-lasting functional impairment (14). Furthermore, macular and peripapillary vessel density was shown to be reduced in advanced stages of glaucoma (15). Also, hemodynamic changes, such as increased blood flow in the optic nerve head, have been reported in early stages of the disease. Remarkably, a linear decline of retinal blood flow occurs during disease progression in experimental glaucoma (16).

#### 1.1.5. Age-related macular degeneration

Age-related macular degeneration (AMD) is an irreversible and progressive neurodegenerative disease that is also a leading cause of blindness in the world with a prevalence of 170 million patients worldwide (17, 18). Degenerative changes in retinal photoreceptors, retinal pigment epithelium (RPE), and Bruch’s membrane (BM) occur especially in the macula leading to central vision loss (18). It is a multifactorial disease and the main risk factor is age. But also impaired retinal and choroidal perfusion is discussed to be part of the pathophysiology (18). The macular part of the retina is characterized by high oxygen consumption (19), and tissues with high oxygen metabolism show elevated generation of reactive oxygen species (ROS). Therefore, the central retinal and choroidal vasculature is more exposed to oxidative stress (18). In eyes with advanced disease stages of AMD a reduced vessel density in the choriocapillaris and hypoperfusion in degenerative areas was found (20). Approximately 10–15% of patients with AMD develop a neovascular disease stage where new fragile choroidal blood vessels grow through the BM to the subneurosensory retina or the sub-RPE with consequent exsudation and further vision loss (21). Choroidal vascular dysfunction is supposed to play a central role in this process. Endothelial dysfunction of the choriocapillaris could be the first step leading to wet AMD. By loss of the vascular support of the RPE angiogenetic signals occur (22). Hence, a higher expression of VEGF was found in eyes with choroidal neovascularization (23). But also retinal vascular changes seem to be part of AMD. A lower arteriole-to-venule ratio, with narrower arterioles was found in eyes with geographic atrophy (24). Furthermore, reduced retinal vessel density was observed in exsudative AMD compared to non-exsudative disease stages (25), indicating that not only the choriocapillaris, but also retinal vessels are affected by disease progression.

#### 1.1.6. Retinopathy of prematurity

Retinopathy of prematurity (ROP) is a disease of premature infants with impaired vascular development and represents a leading cause of childhood blindness worldwide (26). In humans, retinal blood vessels start to develop during gestation, and vascularization of the retinal periphery finishes just before birth (26). In preterm births, the incomplete vascularized retina shows impaired development of the retinal vasculature. By postnatal exposure to a high-oxygen milieu with fluctuations of oxygen levels and consequent generation of ROS endothelial cells of newly formed vessels get injured with consequtive capillary constriction. Delayed physiologic retinal vascular development causes hypoxia with consequent ischemia that leads to angiogenic signaling with increased expression of VEGF. Hence, vasoproliferation with disordered proliferation into the vitreous occurs (26–28).

Altogether, these findings highlight the relevance of vascular anatomy and function for scientific pathophysiological investigations as well as for clinical judgment and treatment of vascular ocular diseases.

### 1.2. Retinal circulation

**FIGURE 1:**
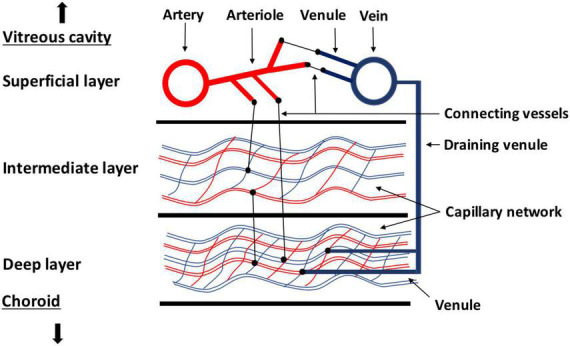
Sketch of the different layers of retinal circulation. In the superficial layer, larger vessels, such as arteries, arterioles, and big veins are located. The capillary network is located in the intermediate and deep plexus with a higher vessel density in the deep plexus. Collecting venules are rising from the deeper plexus to the superficial plexus to drain to the larger veins.

The retina is supplied by two different vascular beds, namely the retinal circulation for the inner retinal part and choroidal vessels supplying the outer retinal part *via* diffusion. Unlike the choroid (29), retinal vascular tone and blood flow are adjusted by autoregulation by non-nervous mechanisms like humorally released local vasoconstrictors and vasodilators (30, 31). The retinal vascular bed can be divided into a superficial, an intermediate and a deep layer. In the superficial layer, larger vessels such as arteries, arteriolar and venular branches and veins are located. The intermediate and deep layer contain the capillary network with the highest linear vessel density in the deep layer. Collecting veins are rising from the deep layer to the superficial layer to drain to the larger veins (32, 33). A sketch of the different layers of retinal circulation is presented in Figure 1. Knowledge on the individual characteristics is important for performing vascular studies. Functional hyperemia induced by flicker light stimulation occurs more pronounced in arterioles of the superficial layer. This phenomenon could be explained by decreasing alpha-smooth muscle actin (α-SMA) levels with increasing vessel size (33). The lowest blood flow and pO_2_ level has been measured in the intermediate layer. Flicker light was reported to produce the highest increase of blood flow in the intermediate layer, which supplies neuronal cell bodies and synapses of the inner retina (33). Light stimuli evoked an increase of pO2 in the outer retina but had no effect on inner retinal layers. During light stimulation, the inner retina has a higher oxygen consumption due to activated ganglion cells that is compensated by increased blood flow (34, 35). The crosstalk between tissue and blood takes place in the capillary bed. However, capillaries are affected first by vascular pathologies or disease progression. These changes are sensed by pre-capillary arterioles (32). To understand vascular diseases, detection of changes in blood flow and vessel diameter especially in smaller vessels is essential.

There are various methods to determine retinal blood flow, but there is still no gold standard. However, for exact measurement of retinal blood flow, reliable determination of vessel diameter and retinal blood flow velocity is necessary. Several studies in humans have already described different methods of measurement. To gain further knowledge on the retinal vascular system and on vascular diseases, studies in animal models are necessary, including genetically modified animals. Particularly challenging is the very small retinal vessel size in rodents, especially in mice, requiring high optic resolution. In this review, we recapitulate different methods for measurement of retinal blood flow and vascular reactivity. Moreover, we describe clinical studies in humans, but also in laboratory animals. An overview of the described methods to measure blood flow and vascular reactivity in the retina is shown in Table 1.

**TABLE 1:** Overview of the described methods to measure blood flow and vascular reactivity in the retina.

	Main content	Principle of measurement	*In vivo*/*Ex vivo*	Commercially available
RVA	Vessel diameter	Fundus camera-based imaging technology	*In vivo*	Yes
LDV	Blood flow velocity	Optical Doppler shift	*In vivo*	Yes
LDF	Retinal blood flow	Scattering theory for light	*In vivo*	Yes
Dye-based Angiography	Visualization of anatomic structures	Passage of a fluorescent dye	*In vivo*	Yes
OCTA	Depth resolved angiograms	Detection of intravascular moving particles as an intrinsic contrast	*In vivo*	Yes
Doppler OCTA	Retinal blood flow	Generation of a reflective profile and detection of phase shifts of the back-scattered lights	*In vivo*	Yes
Retinal oximetry	Oxygen saturation of hemoglobin in RBCs	Difference in absorption of light between oxyhemoglobin and deoxyhemoglobin	*In vivo*	Yes
LSFG	Mean Blur Rate (MBR) as relative index of retinal blood flow velocity	Detection of changes in the speckle pattern by reflection of coherent laser light	*In vivo*	Yes
RBC labeling in vivo	Vessel diameter and passage of fRBCs per second	Detection of fluorescently labeled RBCs by ultrafast confocal line scans	*In vivo*	No
Transmitted light Microscopy *ex vivo*	Vessel diameter	Retinal preparation and measurement of retinal blood vessel reactivity by transmitted light microscopy	*Ex vivo*	No

## 2. Methods of measurement

### 2.1. Analysis of ocular fundus images

For exact determination of retinal blood flow and evaluation of vascular reactivity, precise vessel diameter values are required. There are different fundus camera-based imaging technologies commercially available using photography and concomitant image analysis. One system for real time measurement of vessel diameters is the retinal vessel analyzer (RVA, Imedos Systems; Jena, Germany), which is equipped with a fundus camera connected to a computer system that allows for highly reproducible real-time recordings of retinal arterial and venous diameters (36). It has a measurement range of >90 μm, a temporal resolution of 40 ms and a spatial resolution of less than 1 μm (37). This system is confined to the examination of larger vessels with a diameter >90 μm and requires clear ocular media (38, 39). *Via* the video function, functional analysis of retinal vascular reactivity is possible. Using RVA, flicker-induced vasodilatory vessel responses can be measured (40). RVA is a fast and non-invasive method aimed to detect impaired vascular functions in retinal vascular pathologies. For example, a decrease of flicker-induced vasodilatation during hyperglycemia and in patients with insulin dependent diabetes could be shown (41–43). Other cardiovascular risk factors such as obesity also affect endothelial function and retinal arteriolar reactivity to flicker stimulation (44). In patients with increased cardiovascular risk, improvement of retinal endothelial function after exercise interventions could be detected using RVA (45). Recently, retinal vascular function measured by RVA, was determined for the first time as surrogate for cerebral microvascular function (46). Furthermore, retinal microvascular reactivity to flicker stimulation as marker of endothelial function may be a predictor for cardiovascular events (47). Likewise, in patients with glaucoma, impaired flicker-induced dilatation of retinal veins was observed (48, 49). Furthermore, potentially involved mechanisms in retinal vascular reactivity can be analyzed by RVA. Dorner et al. reported that nitric oxide (NO) is important for regulation of basal vascular tone, but also for flicker-induced vasodilatation (50). Inhibition of NO synthase during isometric exercise in healthy humans reduced retinal venous diameter and responsiveness (51). Recently, the application of a prototype of an adapted RVA in laboratory animals was introduced to evaluate neurovascular coupling in the murine retina (52, 53).

### 2.2. Laser doppler velocimetry

Laser Doppler Velocimetry (LDV) is based on measuring blood flow velocities in retinal arterioles and venules. The optical Doppler shift, i.e., the differential reflection of light according to blood flow velocity, is directly proportional to blood flow velocity when the vessel of interest is illuminated with a high coherent laser beam. The flow velocities within the vessel of interest correspond to the range of frequency shifts of the reflected laser light. However, the maximum frequency shift corresponds to the maximum center velocity within the vessel (38, 54). To receive absolute values of red blood cell (RBC) velocities in individual retinal blood vessels, bidirectional LDV is applied by assessing Doppler-shift frequency spectra for two directions of the scattered light. The differences in cutoff frequencies, which are directly related to the top speed of RBCs [V(max)], are utilized to obtain absolute V(max) values (55). Combination of bidirectional LDV with retinal vessel diameter measurement from monochromatic fundus photographs or from RVA allows for calculation of retinal blood flow in a single vessel (56, 57). Previous studies in humans assessed total retinal blood flow by summing up data from all vessels with a diameter of >60 μm entering the optic nerve head (57). The Canon Laser Doppler blood flowmeter (CLDF, Canon; Tokyo, Japan) combines the technique of LDV for blood speed measurement with a retinal diameter assessment system (58). Based on the Poiseuille principle, it is possible to calculate retinal blood flow in μl/min in a selected vessel with high reproducibility (59).

Vascular reactivity can also be evaluated by performing LDV. By using bi-directional LDV in combination with the RVA, increased retinal blood flow due to diffuse luminance flicker has been shown (60). Impaired flicker-induced retinal vasodilatation during exposure to cardiovascular risk factors, such as smoking, was also demonstrated using this method (61).

### 2.3. Laser doppler flowmetry

An advanced technology utilizing laser light to determine retinal blood flow is Laser Doppler Flowmetry (LDF). The coherent laser light is directed on vascularized tissue where no larger vessels are visible. This technique is based on the scattering theory for light in tissue formulated by Bonner and Nossal (38, 62). Scattering on moving red blood cells (RBCs) causes a frequency shift in the scattered light. Relative measurements of the mean velocity of RBCs and blood volume is obtained by complete randomization of light directions impinging on the RBCs. The product of velocity and volume gives relative values of blood flow (38, 63). Vascular reactivity during different stimuli can be analyzed by combination of RVA and LDF. It has been shown that short-term increase of intraocular pressure does not influence the response of retinal vessel diameters and optic nerve head blood flow during flicker-stimulation (64). This method was also employed to determine optic nerve head blood flow. Isometric exercise is associated with increasing ocular perfusion pressure by autoregulation of optic nerve head blood flow (65, 66). Limitations are the varying scattering properties between individuals due to varying vessel densities and vessel orientation within the small volume of tissue sampled by LDF. Thus, interindividual comparison of LDF data is difficult and not recommended (38). Due to high intraindividual reproducibility (67), LDF can be used for studies on responses to physiological and pharmacological stimuli (38). During measurement of retinal blood flow contribution from the underlying choriocapillaries to the signal cannot be excluded (68). Determining very high or low velocities can be hampered by the limited frequency range used. As capillary retinal blood flow is pulsatile and, thus, deviations due to different phases in the cardiac cycle are common, recording should be triggered by electrocardiography (68). Another source of interference is caused by a displacement of the analysis window with the consecutive measurement of different geographic areas (68).

Scanning laser Doppler flowmetry includes the principle of scanning laser tomography. The commercially available Heidelberg Retina Flowmeter provides a two-dimensional flow map of the retina and the optic nerve head and allows for reproducible evaluation of capillary blood flow (69). A previous study showed good correspondence of scanning LDF in rats with angiograms and resolution to third order arterioles and venules. But neither superficial nor deep capillary circulations could be sufficiently visualized. Hence, the technique of scanning LDF is limited to larger retinal vessels (70).

### 2.4. Dye-based angiography

Dye-based angiographic techniques are commercially available and allow for visualization of anatomic structures by the passage of a fluorescent dye. There have been different approaches to evaluate blood flow velocity. By measuring the time required for the dye to pass through retinal circulation, the mean retinal circulation time defined as the difference between venous and arterial times is determined (38). Alternatively, the determination of the arterio-venous passage time defined as the time between the first appearance of the dye in a retinal artery and in the corresponding vein (71), is applied. This concept assumes that the absolute blood flow in one defined area supplied by one specific artery is drained into one corresponding vein. Obviously, this approach cannot work in patients with vascular diseases (38). As dye-based angiography does not provide sufficient depth resolution to fully evaluate the retinal vasculature, only the superficial vascular plexus can be explored. The visibility of capillaries decreases rapidly with increasing distance from the foveal center, especially for deeper capillary circulation (72). Furthermore, capillary visualization rapidly decreases with capillary size. Only 40% of capillaries <4,5 μm were visible on fluorescent angiography (73). To visualize capillaries with higher sensitivity, adaptive optics such as adaptive optics scanning light ophthalmoscopic fluorescein angiography have been developed (74). Of note, dye-based angiography is invasive since it requires intravenous dye administration, which may cause side effects up to rare but serious anaphylactic reactions (75, 76). An argument in favor of fluorescein angiography is the dynamic information obtained, such as identifying dye leakage in case of disruption of the BRB (1, 73). Further, it captures large areas of the fundus in one single image providing an overview of retinal vascular pathologies such as leakage, microaneursyms, areas of non-perfusion or retinal neovascularization (1).

### 2.5. Optical coherence tomography angiography

**FIGURE 2:**
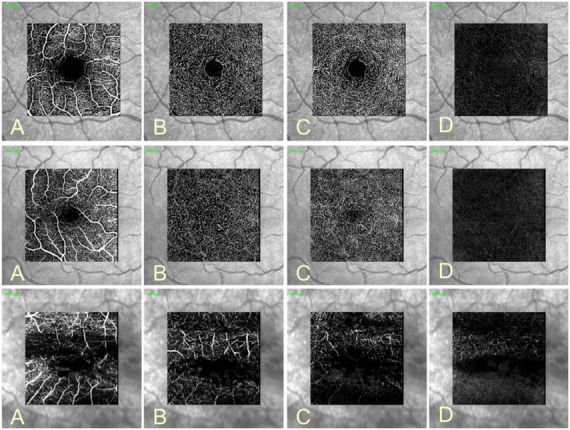
Optical coherence tomography angiography (OCTA) images of a healthy right eye (upper row), ROP (middle row), and RVO (lower row). OCTA detects moving particles within vessels as intrinsic contrast and generates angiograms with depth resolved images of the retinal vasculature. With this method, the microvasculature of the superficial **(A)**, the intermediate **(B)**, the deep vascular plexus **(C)** and the avascular plexus **(D)** can be shown. Projection artifact removal (PAR) uses information of the superficial vascular plexus to remove artifacts from OCTA images. In the middle row the typical reduction in size of the foveal avascular zone in ROP patients can be seen. The lower row shows OCTA in RVO. Due to macular edema segmentation of retinal layers is aggravated with consequent displacement of retinal vascular layers.

Optical coherence tomography angiography (OCTA) is a commercially available method, that detects intravascular moving particles as intrinsic contrast and produces angiograms by comparing motion-related differences between repeated OCT-B-scans taken exactly at the same location. Sedentary structures generate similar signals (correlated signals), while moving intravascular blood cells produce different OCT signals from one scan to the other (decorrelated signals). These different signals are translated to blood vessels in the angiograms delivering depth-resolved images of the retinal microvasculature, which come close to histological pictures (1, 73). Some exemplary OCTA images of the retinal vascular plexus in a healthy right eye and in eyes with vascular diseases such as ROP and RVO are shown in Figure 2. OCTA provides the possibility to analyze different features of retinal vascular pathologies such as diabetic retinopathy including microaneurysms (1), impaired vascular perfusion, neovascularization in various retinal layers (77), non-perfusion areas or cotton wool spots (78). Hence, for some diseases, OCTA represents a feasible non-invasive alternative for the frequently used fluorescein angiography. In addition, quantitative measurements such as perfusion density (vessel area = percentage of the area occupied by a vessel) or vessel density (vessel length = total length of skeletonized vessels in a certain area, mm/mm^2^) can be acquired (79–82). In addition, parameters for specific questions such as the vessel diameter index, the fractal dimension (branching complexity of the capillary network), the intercapillary area, the vessel length fraction or the vascular architecture, e.g., branching angles, can also be determined (1). The development of OCTA-based automatic segmentation algorithms provides the possibility to quantify and to evaluate capillaries and large vessels separately (83). Analysis of macular parafoveal vessel density in different capillary layers, vascular reactivity to different stimuli as hypoxia or isometric exercise causing vasodilatory or vasoconstriction responses could be performed (84). By performing OCTA it was possible to access microvascular reactivity in different retinal vascular layers. During the transition from light to dark an increase of vessel density in the superficial capillary plexus and a decrease in the intermediate and deep capillary plexus was found. Furthermore, a significant increase of vessel density in the superficial capillary plexus and a decrease of skeletonized density was found during flicker stimulation, indicating an active dilatation of the larger vessels with following redistribution of blood flow from the smaller capillaries to the larger vessels of the superficial capillary plexus (85). Other groups reported that vessel densities in superficial macular regions determined by OCTA were reduced in eyes of glaucoma patients (86–88). The degree of vessel rarefication also correlated with the loss of visual field and disease severity (89). Experimental acute elevation of intraocular pressure over 10 mmHg showed a decrease of choriocapillary blood flow, and elevation of intraocular pressure over 20 mmHg reduced retinal vessel density in OCTA indicating that autoregulation of retinal microcirculation becomes impaired by elevation of intraocular pressure (90). Studies in small laboratory animals have rarely been described so far. One experimental study could clearly visualize the normal vascular plexus in different retinal layers using OCTA in mice. Additionally, an *in vivo* evaluation of laser-induced choroidal neovascularization has been shown (91). The measurement of retinal vessel density is valid and reproducible indicating that OCTA is qualified for longitudinal assessment of vascular changes in various mouse models (92). A diminished vessel density in mice with type 1 diabetes has also been observed by the use of OCTA technology. Because of precise depth information, it was possible to separate each vessel layer (93). Finally, OCTA imaging in mice is superior to confocal microscopy for detecting structural details in the deeper vascular network (94).

These studies indicate that OCTA is a valuable non-invasive method to visualize the retinal vasculature with depth resolution *in vivo* providing the information of depth localization of vascular pathologies (73). By software based image processing, it is possible to generate quantitative markers for vascular pathologies and additional volumetric data can be segmented (73). Furthermore, the images are well defined, highly contrasted and not obscured by hyper fluorescence from dye leakage (1). In contrast, OCTA cannot access alterations in vascular permeability or leakage. Coexisting macular edema, for example in diabetic retinopathy, can hamper proper segmentation of retinal vascular layers. Quantification of blood flow in OCTA is difficult because it only detects flow between a minimum and maximum threshold (1). Furthermore, OCTA images are highly dependent on the OCT instrument, scan protocols and signal processing, making comparisons between different devices difficult (73). Several types of artifacts such as localized loss of signal strength by vitreous floaters, eye movement or movements within the eye due to pulsatile expansion of the choroid can be sources leading to misinterpretations (95, 96). In studies on small laboratory animals, mouse motion and heart beats may also affect image acquisition (93). As OCTA detects vessels by particle motion, it does not visualize vessels without flow (93) and the detection rate of vessels with slow blood flow such as certain subtypes of microaneurysms decreases (97–99). The determination of the vessel diameter is an important feature to analyze retinal blood flow. It has been shown that the diameters of retinal vessels measured from OCTA scans were generally wider than with measurements obtained from adaptive optics ophthalmoscope (AOO) (100) or fundus photographs (101) regardless of scan protocols and vessel types. Reasons could be the oversampling rate of the OCTA system, the poorer lateral resolution compared with the AOO system or the effect of binarization (100). The measurement could also be affected by the intraluminal presence of a cell free plasma layer whose width is strongly correlated with the vessel diameter (102). Others compared OCTA and high resolution confocal microscopy perfusing porcine eyes with red blood cells. In these studies, a good representation of retinal vessels with larger caliber, but an under representation of microvessels smaller than 10 μm and branch points in all four retinal plexuses, particularly the intermediate capillary plexus, was observed. Also, the sensitivity to detect vessels was reduced with increasing retinal depth (103). A reason for weaker OCTA signals in vessels with a diameter under 100 μm could be a hemodynamic feature, called Fahraeus-Lindquist effect. It leads to a diameter-dependent decrease of hematocrit and effective blood viscosity, which means that there are less blood cells and larger plasma gaps in the smaller retinal capillaries (103). Moreover, the fact that the OCTA image signal is produced by red blood cell movement within the vascular lumen may also explain that the vessel diameter measured by OCTA does not exactly correspond to the actual size. The pixel resolution of OCTA images is approximately 3.85 to 4.14 μm per pixel suggesting that slight changes in vascular diameter may remain undetected by OCTA (103). Especially in small laboratory animals such as mice with retinal arteriole diameters from 10 to 40 μm, only diameter changes of ≥10% can be detected with this spatial resolution.

Due to the large amount of data that has to be evaluated in the context of OCTA recordings, various algorithms have been developed for automated evaluation. One of the most studied parameters of OCTA is the foveal avascular zone (FAZ). Numerous methods for quantifying FAZ changes with OCTA have been investigated, including horizontal and vertical diameter, two-dimensional total area, remodeling, acircularity index (AI), and axial ratio (104–107). Because there is not yet a consensus on how best to quantify FAZ with OCTA, comparing the clinical utility of FAZ metrics with other measures of DMI remains difficult. In addition, there is considerable variability in the size and shape of FAZ in patients (108), which may complicate FAZ measurements. Furthermore, many studies have considered FAZ in segmented vascular layers, even when the individual vascular plexuses fuse at the fovea, leading to conclusions about FAZ size that are related to segmentation technique rather than pathologic vascular changes.

**FIGURE 3:**
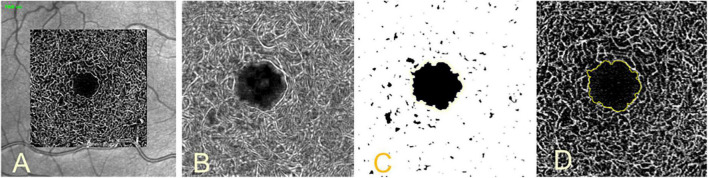
Segmentation of the foveal avascular zone (FAZ). Image of the intermediate vascular plexus image **(A)**, same image after noise reduction, zoom and vessel-amplification using Gabor edge filters **(B)**, isolated FAZ after thresholding segmentation **(C)**, segmented FAZ in yellow outline in unedited image **(D)**.

One of the most important factors affecting FAZ measurements is the segmentation of the FAZ boundary. While several reports (109–111), have demonstrated high repeatability of semiautomated FAZ detection with commercial OCTA systems, some studies (112, 113) have found substantial differences between FAZ detection algorithms and manual delineation. Accurate FAZ segmentation becomes even more challenging with DR because irregular delineation can lead to algorithm failure and inappropriate inclusion or exclusion of abnormal FAZ sections. Figure 3 shows a not yet published algorithm of the University Eye Hospital Mainz to automatically segment and measure the FAZ in OCTA images. To achieve this, the noise in the images is first reduced and then the signal from the vessels is amplified using Gabor edge filters. The image is then segmented with the Li’s Minimum Cross Entropy thresholding method (114) to isolate the FAZ. Afterward the size of the isolated FAZ is measured.

### 2.6. Doppler optical coherence tomography

Optical coherence tomography can be expanded to Doppler OCT by generating a reflective profile and by detecting phase shifts of the back-scattered lights, which are caused by mobile red blood cells within the analyzed volume (115, 116). It can provide fully quantitative volumetric information on blood flow and vascular and structural anatomy (115). The velocities in a Doppler OCT require a certain range of Doppler angles between blood flow and the scanning laser beam, called angle bandwidth, which is dependent on the absolute blood velocity within the vessel. To obtain absolute blood flow velocity, it is necessary to know the angle (115). Different techniques have been used to determine the angle from consecutive images, however, all of them were strongly interfered by eye movements (117). Single beam approaches showed high angle error, especially when the angle of incidence changed because of eye movements (118, 119). Others resolved the problem of the *a priori* unknown Doppler angle by multiple beam illumination. For absolute flow measurements, a bi-directional technique has been used *in vitro* by illuminating the sample from two angles with orthogonally polarized beams (120). Unfortunately, this approach was not feasible to measure human retinal blood flow. By illumination of the sample with two linearly and orthogonally polarized beams separated by their polarization properties and incident at a known angle on the sample, determination of retinal blood flow independent of the angle of incidence was possible (116, 121). By the use of a simple geometric arrangement, absolute blood velocity can be measured when the angle between both beams is known (122). These measurements of total retinal blood flow have shown excellent reproducibility and can be used for longitudinal studies (122). Studies in glass capillaries *in vitro* have shown good agreement between dual-beam bidirectional Doppler OCT measurements and perfusion rates (116). Some authors conducted Doppler OCT measurements in diabetic patients with or without diabetic retinopathy and in patients with other ocular pathologies such as glaucoma or branch retinal vein occlusion and observed reduced total retinal blood flow (123, 124). Flicker light-induced hyperemia was shown by dual-beam bidirectional Doppler OCT (125, 126). During 100% oxygen breathing a significant decrease in retinal blood flow has been shown by using a dual-beam bidirectional Doppler FD-OCT, resulting in a decrease in retinal oxygen extraction (127). Studies in laboratory animals have not been described so far. Doppler OCT is a non-contact and label-free method to determine retinal blood flow. Moreover, it delivers quantitative volumetric information on blood flow with vascular anatomy and angiographic information in the same data set (115). There are also several limitations to be considered. First, the minimum detectable velocity in Doppler OCT depends on the scan rate. Hence, faster image acquisition could reduce velocity sensitivity (115). Another critical issue are eye movements changing the Doppler angle. Notably, changes of only some degrees will produce incorrect results (115).

To determine total retinal blood flow, all retinal blood vessels that enter the optic nerve head need to be analyzed by adding all flow values in arteries or veins to calculate total blood flow (115). Because blood flow fluctuates during the cardiac cycle, the measurements need to be averaged over several heart beats (115). The values can be obtained from arteries or veins only or from both as internal control (115). However, for volumetric blood flow calculations, the vessel diameter needs to be known. Variations in vessel diameter are challenging as they enter the blood flow calculation quadratic (115, 128). Vessel diameter extraction from OCT images is problematic because the transversal OCT resolution is insufficient unless not combined with adaptive optics systems. Moreover, blood is highly scattering and absorptive, which produces shadowing effects behind the vessel and obscures the rear vessel boundary (115). There is an erythrocyte-free region consisting of plasma only close to the vascular wall from which no Doppler signal is obtained. Low velocities at the border of the vascular wall could lead to underestimation of vessel diameters (115). To address these problems some authors obtained vessel diameters from fundus photographs (116) or retinal vessel analyzer (129) separately.

### 2.7. Retinal oximetry

Retinal oximetry requires metabolic imaging for diseases of the retina by measuring oxygen saturation of hemoglobin in RBCs (130). It has been shown that oxygen metabolism is disturbed in several eye diseases. This is especially true for ischemic retinal pathologies, such as diabetic retinopathy and retinal vein occlusion, where abnormal oxygen metabolism is a main part of the pathophysiology. In atrophic retinal diseases, such as glaucoma or retinitis pigmentosa with reduced oxygen consumption this information is also clinically relevant (130). Non-invasive retinal oximetry is based on the difference in absorption of light between oxyhemoglobin and deoxyhemoglobin. The reference wavelength, so called isobestic wavelength, is defined as the wavelength where absorption qualities are equal for hemoglobin bound and unbound to oxygen. In contrast, the non-isobestic wavelength is sensitive to changes in the oxygen saturation (130). The optical density (OD) specifies the light absorbance of a solution, for example blood. The ratio of this optical density between both wavelengths, the so called optical density ratio (ODR), is approximately linearly related to the oxygen saturation (131, 132). Two different systems are commercially available at present. The Oxymap oximeter (Oxymap ehf, Reykjavik, Iceland) employs two cameras and a beam splitter. It captures simultaneously two images at an isobestic and non-isobestic wavelength (570 and 600 nm) to estimate retinal vessel oxygen saturation (133). The Imedos oximeter (Imedos, Jena, Germany) is composed of a fundus camera with a special dual wavelength transmission filter and a color-charge coupled device camera that records two monochromatic fundus images at 548 and 610 nm. The oxygen saturation is obtained by calibration of ODR measurements of healthy control subjects (134). Both systems are commercially available and display excellent repeatability. However, because of an insufficient concordance of arterial oxygen saturation data between both devices, patients should be followed intra-individually by one device only. These differences are due to different absorbance wavelengths and by different image post-processing algorithms used (135). In a large cohort covering low and high ages, a retinal arterial oxygen saturation of 92.2 ± 3.7% and retinal venous oxygen saturation of 55.5 ± 6.3% was reported (133). Due to sparse vascularization and high retinal oxygen consumption the arteriovenous decrease of about 35% is high compared to other tissues (130). Different retinal vessel oxygen saturations were detected in the retinal quadrants with the lowest oxygen saturation in the inferotemporal quadrant (133, 136, 137). This shows that retinal oximetry can examine regional features, but it remains to be clarified whether they are real or artificial. Regional differences in measured saturations could be due to technical artifacts. Saturation can vary, when the angle of gaze is changed due to the fact, that the light bundle entering the eye is now oblique impacting on the distribution how the retinal area observed is illuminated (138). Regardless to the reasons, quadrant differences should be kept in mind when analyzing retinal oximetry. There are further limitations inherent to retinal oximetry. The available systems are limited to arterioles and venules above a certain diameter and are not capable to measure capillaries. The Oxymap T1 can generate highly reproducible data in vessels wider than 56 μm. The variability of measurements increased with narrower vessels (130). But capillary levels can also be studied by analyzing venous oxygen saturation because the oxygen delivery from the capillary to the tissue is reflected by the arterio-venous difference in oxygen saturation (130). Age can also complicate retinal oximetry as retinal oxygen saturation is affected by reduced retinal blood flow and consecutive decrease of oxygen extraction. Geirsdottir et al. showed a slightly decrease of retinal venous oxygen saturation with increasing age while retinal arterial oxygen saturation remained unchanged (133). Others demonstrated a decreasing retinal arterial and venous oxygen saturation (139). Due to decelerated blood flow, oxygen can be underestimated by diffusion through the vessel wall into the interstitial compartment. The determination of retinal oxygen saturation can also be complicated by poor image quality due to unclear optical media (130). Other individual factors such as ocular perfusion pressure (133), personal finger oximetry values (140), hypertension (139) or myopia (141) affecting oximetry measurements may influence precision. Systemic hyperoxia or hypoxia also affect retinal oxygen saturation (130). In hyperoxia, oxygen saturation increases in retinal arterioles and even more in venules and induces vasoconstriction thereby affecting retinal oxygen saturation. Due to oxygen flow from the choroid, less oxygen is extracted from the retinal circulation and oxygen saturation especially in venules increases (142). In hypoxia, a smaller decrease of oxygen saturation in venules than in arterioles was observed (131, 143). This phenomenon could be due to hypoxic vasodilatation in arterioles to counteract the reduced oxygen concentration (144). Retinal oxygen demand is also influenced by light conditions changing the metabolic rate of the retina (130). Oxygen saturation is increased in arterioles and venules when transitioning from light to darkness (145). Compared to steady light conditions, oxygen saturation is elevated during flickering light (146). It is known that retinal photoreceptors use more oxygen in the dark than in steady light (34). Darkness and flickering light are both believed to increase the retinal metabolic rate compared to steady light (130). Moreover, an increment in retinal blood flow is associated with a higher retinal oxygen extraction (147). Although there are numerous factors affecting retinal oxygen saturation, retinal oximetry offers a useful tool for disease monitoring *in vivo* as for example in retinal vein or artery occlusion, diabetic retinopathy, age-related macular degeneration or retinitis pigmentosa (130).

To obtain quantitative data on oxygen extraction, retinal blood flow needs to be measured together with the arterio-venous oxygen difference. In hyperoxia and in hypoxia a reduced arterio-venous oxygen difference was detected (148, 149). In hyperoxia, retinal blood flow is also reduced and retinal oxygen extraction decreases because large amounts of the choroid are diffusing to the inner retina (148, 150). In hypoxia, blood flow is increased and oxygen extraction is constant (149). Based on these findings, it is necessary to combine retinal oximetry with retinal blood flow measurement. The combined retinal vessel oximetry with bidirectional Doppler OCT, as described above, presenting a method with high potential to study oxygen metabolism in hypoxic retinal diseases such as diabetic retinopathy (127). Based on a mathematical model, oxygen extraction is calculated (127). This method has also some limitations. The oxygen extracted from retinal blood vessels is not necessarily equal to the oxygen consumed by the inner retina, since a part of the oxygen supplied by retinal vessels also oxygenates the photoreceptors. This technique determines total retinal oxygen extraction indicating that the evaluation of local hypoxia is not possible. Under some conditions, such as 100% oxygen breathing, the choroid may contribute to the oxygenation of the inner retina (130, 150).

### 2.8. Laser speckle flowgraphy

**FIGURE 4:**
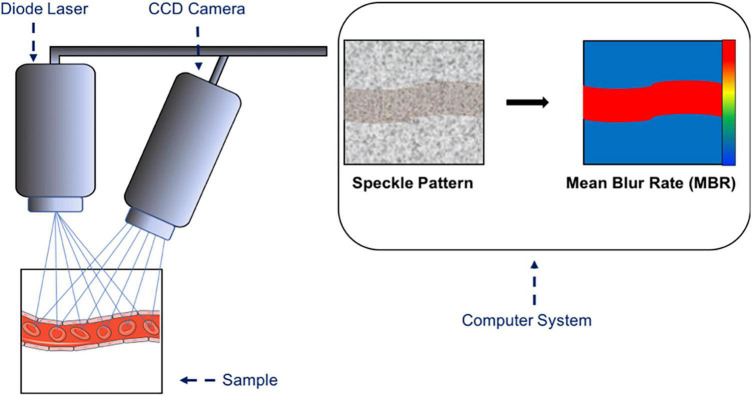
Sketch of the principle of laser speckle flowgraphy. A microscope equipped with a diode laser is coupled to a charge-coupled device (CCD) camera. The laser light is reflected by the irregular surface of the fundus and is translated into a speckle pattern of the scattered light. The mean blur rate (MBR) is calculated from the speckle pattern and is a relative index of blood flow velocity in arbitrary units. The highest MBR is shown in red and the lowest MBR is shown in blue.

Laser Speckle Flowgraphy (LSFG) utilizes changes in the speckle pattern of coherent laser light, which is reflected by the irregular surface of the ocular fundus. The speckle pattern contrast results in the mean blur rate (MBR), which is linearly proportional to blood flow velocity and microsphere-derived volume blood flow (38, 151, 152). The technique is commercially available and requires a fundus camera or microscope equipped with a diode laser (wavelength, 830 nm) and a charge-coupled device (CCD) camera (151, 153, 154). A sketch of the principle of measurement is demonstrated in Figure 4.

Retinal blood flow can be quantified by two methods, the annulus-defined method and the Total Retinal Arteriole and Venule Analysis (TRAVA). By using the annulus-defined method, a large annular area of the retina outside the optic nerve head needs to be defined, and total retinal blood flow within the included retinal arteriole and venule segments is measured. By using TRAVA, blood flow within individual vessels is determined. The user defines the midline of a certain vessel and the length of the area measured (151). A good agreement between these two methods has been reported. The lowest intra- and inter-session variability has been shown for the less time-consuming annular method (151). TRAVA is more time consuming and more dependent on the definition of vessel borders by the operator. Therefore, the annular method has lower computation time, inter-operator variability and inter- and intrasession variabilities. However, TRAVA allows for distinct analysis of arterioles and venules, which is necessary for studies of vessel-specific diseases, e.g., branch retinal venous occlusion (155). To detect changes in the vessel diameter caused by vascular constriction, dilation or remodeling, the “percent vessel area”, a surrogate parameter of vessel width, was developed. With this method, the number of “white” pixels above a given threshold is measured. By using a defined threshold and maintaining a constant total sample pixel area by using the same annulus in each image, a reduction in percent vessel area suggests a decreased luminal diameter (151).

Several studies utilized LDFG to quantify retinal blood flow under physiological conditions and in several ophthalmic diseases in humans. For example, LSFG has been used to examine flicker light-induced hyperemia in the retina and the optic nerve head (156). Moreover, a good correlation of LSFG data with absolute blood flow measurements obtained from Doppler-OCT has been reported with high reproducibility of the LSFG system (157). Also, longitudinal changes of ocular blood flow were monitored using LSFG (158). In various ocular pathologies, such as glaucoma or diabetic retinopathy, this method delivered also highly reproducible data and the possibility to measure changes of blood flow by pharmacological intervention (159, 160). In patients with vascular risk factors, LSFG has been utilized to examine pressure autoregulation of optic nerve head vessels (161). Recently, the LSFG-Micro (Softacare, Fukuoka, Japan) instrumentation was introduced, which offers the possibility to measure blood flow in small animals. For example, longtime studies in rats over a period of several months were reported to provide reliable information on longitudinal changes in optic nerve head blood flow (162). By determining different parameters of blood flow such as MBR of total area (MA), vessel region (MV), and tissue region (MT) regional differences in blood flow could be found (162). There have also been reports of parallel studies in different groups providing the possibility to compare blood flow in physiologic and pathological animal models in longtime studies (163). The monitoring of pharmacological interventions in rats has also been studied using LSFG (164). Studies using LSFG in mice are very limited so far (151, 165–168). Also in mice, LSFG-Micro was reported to show consistent and reproducible retinal blood flow measurements for non-invasive monitoring of vascular functions in the retina (166). Moreover, LSFG was applied in a transgenic mouse model developing diabetic retinopathy (165). Others could monitor the effects of pharmacological interventions on retinal venous blood flow in mice (168). Since recently, a new protocol for quantifying flicker-light-induced hyperemia in mice with low variability over various imaging sessions is available. Hence, LSFG may be applied in serial studies (167). Others also demonstrated significant elevation of retinal blood flow during flicker stimulation and a significant decrease of retinal blood flow during systemic hyperoxia (166). These studies present LSFG as a novel minimally invasive tool to measure retinal blood flow in small laboratory animals and in humans providing the opportunity to conduct parallel studies on retinal blood flow in humans and relevant animal disease models. The method requires minimal time effort for recording and post-processing and is commercially available constituting an easy alternative to Doppler OCT, which requires various OCT detection beams addressing the major limitation of Doppler angle-dependent velocities (151). There are also several limitations using LSFG. When comparing ocular blood flow between individuals several systemic factors, e.g., blood pressure, age or sex and ocular factors, such as axial length, should be adjusted (154, 169). The measurement of MBR may also be affected by changes in IOP (169). The use of LSFG in laboratory animals requires anesthesia, which may also influence retinal blood flow measurement (170). To meet this problem, the anesthetics used should be taken into consideration during design and analysis of functional blood flow studies (151, 170). Moreover, evaluation of vascular stiffness and remodeling is difficult in small laboratory animals, because their high physiologic heart rate complicates the measurement of dynamic blood flow changes during systole and diastole.

Furthermore, the amount of pigment in the retinal pigment epithelium has an influence on the intensity of the choroidal LSFG signal. Therefore, in animals with a dark fundus pigmentation the deeper choroidal flow signal may not be detected, whereas in animals with slight fundus pigmentation retinal blood flow measurement may be interfered by the contributing signal of the choroid (151).

### 2.9. Red blood cell labeling *in vivo*

Retinal blood flow measurement can be facilitated by fluorescent labelling of red blood cells (fRBC). Recently, an *in vivo* technique has been introduced, by which ultrafast confocal line scans capture fRBC in the rat retina (33, 171, 172). First, rats are anesthetized and paralyzed to reduce eye movement. To label red blood cells, arterial blood is withdrawn and erythrocytes are isolated by centrifugation. The cell solution is then mixed with the dye solution and re-suspended in blood plasma solution. The suspension is re-injected into the venous line and a fRBC density of about 0,9% in large vessels and 1,2% in capillaries is reached. To visualize the vascular wall, fluorescent isothiozyanate (FITC) dextran is injected intravenously. Vascular diameter and fRBC flow can now be measured at the same time by using confocal line scans orientated perpendicularly to the vessel. Illumination by the laser needs to be kept at a minimum to limit photoreceptor stimulation. The diameter of a blood vessel is calculated from the distance between the vascular wall borders and flux is measured by counting the passage of single fRBCs per second (RBS/s). For calculation of vessel diameter and flux values the MATLAB program can be used, which calculates the vascular diameter by automatic extraction the inner vessel borders and by removing light artifacts. To measure fRBC flow, MATLAB detects fRBCs from the background by the use of a moving threshold algorithm. Assessment of blood flow in the retina was achieved by two different mathematical arrangements using flow, RBC/volume ratio and RBC/fRBC ratio or velocity and luminal cross-sectional area. This method allowed for measurement of flicker light-induced vasoreactivity in the rat retina, where resulting hyperemia was reported to be more pronounced in arterioles than in downstream capillaries and venules (33). Likewise, blood flow changes during hypoxia and hyperoxia were measured in retinal vessels of various sizes (171). Other authors performed red blood cell labeling to measure retinal blood flow as well. For example, in diabetic mice with persistent hyperglycemia a higher velocity of FITC-labeled red blood cells was observed (173). By using scanning laser ophthalmoscopy, hemodynamic differences in FITC-labeled normal and sickle-RBCs could be visualized (174). Moreover, follow-up studies on retinal red blood cell velocities have been performed with fluorescently labeled RBCs in growing mice (175). Others conducted measurements of retinal blood velocities and vessel diameter changes by tracking fluorescent microspheres with a scanning laser ophthalmoscope (176–178). Red blood cell labeling provides the possibility to visualize and quantify single red blood cell flow simultaneously in arteries, veins and capillaries in small laboratory animals. In comparison, FD-OCT measurements are limited to vessels with a diameter of >30 μm (179). It is a non-invasive technique, and longitudinal repetitive imaging in the same animal is possible. Moreover, the technique may be useful to monitor progression and treatment efficacy in mouse models of retinal diseases. It is based on simple cell counts, and results were shown to be reliable and accurate (171). But there are also several limitations of this technique. First, the results may be affected by anesthesia, which can have strong effects on blood flow (180). Second, the diameter of venules is underestimated compared to arterioles (171). Third, flux is used as marker of retinal blood flow. However, the calculation includes red blood cell flux only and not the flow of whole blood, which also contains plasma and white blood cells (171). Hence, comparison between vessels is confounded, because the hematocrit is varying in individual vessels and in most capillaries (181). Hematocrit is also affected by the Fahraeus-Lindquist effect describing that hematocrit decreases with decreasing vessel diameter (171). Moreover, measurement of fRBC flux by confocal line scans is limited to only one vessel at the same time and needs high temporal resolution (171). Also, the use of the technique in humans is complicated and invasive because blood collection and reinjection is required.

### 2.10. Transmitted light microscopy *ex vivo*

**FIGURE 5:**
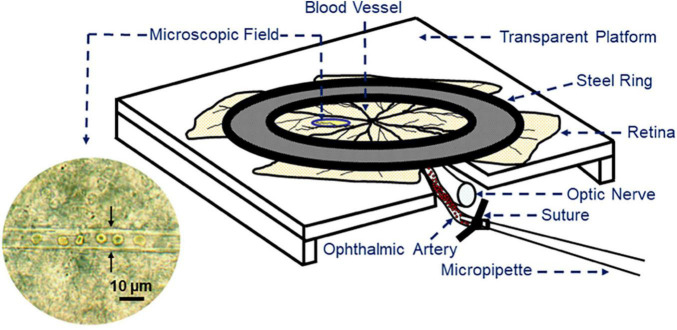
Sketch of the retinal preparation for retinal blood vessel reactivity measurement by transmitted light microscopy. An isolated retina with the optic nerve and the ophthalmic artery is fixed on a transparent plastic platform by a steel ring. To pressurize the retinal vasculature, the ophthalmic artery is cannulated with a micropipette, which is connected to a reservoir filled with buffer solution. In the microscopic field, a retinal arteriole with luminal red blood cells is visible. The arrows point to the vascular walls.

Microvascular analysis of retinal arterioles in laboratory animals is challenging due to small retinal blood vessel diameter (<40 μm). Transmitted light microscopy allows for visualization of changes in mouse or rat retinal vessel diameter with high optic resolution *ex vivo* by a self (182). First, the eye globe is excised with attached orbital tissues followed by preparation of the ophthalmic artery and ligation of their small branches. Finally, the retina is isolated from the eye globe and transferred to a perfusion chamber consisting of a transparent reservoir with an in- and outflow tube, a peristaltic pump and circulating externally oxygenated and carbonated buffer solution. Next, a glass micropipette connected to a silicon tube is inserted into the ophthalmic artery to pressurize the retinal vascular system. After placement of the retina onto a transparent platform, the retina is spread out and fixed to the bottom. The perfusion chamber containing the retina is then placed under a light microscope with a 100x water-immersion lens. A sketch of the setup is shown in Figure 5.

Once, a blood vessel with a good visible wall is found, it can be exposed to various mechanical and pharmacological stimuli. Mouse vessels respond well to the vasoconstrictor, 9, 11-dideoxy-9α, 11α-methanoepoxy prostaglandin Fα (U46619), a thromboxane mimetic, but weakly to α_1_-adrenoceptor agonists, such as phenylephrine (183). Following preconstriction, mouse retinal arterioles show pronounced vasodilatory responses to the endothelium-independent vasodilator, nitroprusside, and to the endothelium-dependent agonist acetylcholine (182). In arteries with damaged endothelium, no vasodilation but pronounced vasoconstriction is seen in response to acetylcholine, indicative of endothelial dysfunction (183, 184). Different risk factors contributing to endothelial dysfunction and impaired autoregulation can be analyzed using this method in mice and rats. For example, elevated intraocular pressure was shown to cause abnormal retinal arteriole function, which may contribute to the pathophysiology of glaucoma (13). It has also been demonstrated that mice exposed to acoustic or chronic social defeat stress develop impaired endothelial function in retinal arterioles (185, 186). Measurement of vascular reactivity in different genetically modified mice offers the possibility to analyze and to compare different phenotypes leading to retinal vascular diseases. For example, apolipoprotein E or endothelial nitric oxide synthase deficiency were shown to cause endothelial dysfunction in mouse retinal arterioles (187, 188).

Additionally, effects of pharmacological interventions on arteriole reactivity have been tested performing transmitted light microscopy in mouse retinal blood vessels. For example, protective effects of betulinic acid following ischemia-reperfusion injury in the mouse retina have been recently reported (189).

The translucent character of the isolated retina enables visualization of retinal arterioles by the use of a transmitted light microscope with high optical resolution up to the Abbe diffraction limit between 200 and 300 nm when white light is used. Therefore, depending on the vessels size, diameter changes of 1–3% are detectable by this technique in the mouse retina (182). In contrast, stereomicroscopic techniques are limited by their optical resolution, which impedes the detection of small changes in vessel diameter when no additional sophisticated devices, such as the Adaptive Optics Scanning Light Ophthalmoscope or fluorescent dyes are used (171, 190). Another advantage of the *ex vivo* setting is the fact that anesthetics or changes in systemic blood pressure have no influence on the measurement, which allows to draw clear conclusions on local mechanisms of vessel diameter regulation. However, a drawback of this self-developed technique is the long preparation time of about 90 to 120 min by trained investigators. When the preparation takes longer than 180 min, endothelial function starts to decrease. Moreover, no more than three consecutive concentration-response curves should be performed in the same retinal preparation because more repetitions may reduce vascular responsiveness. Another disadvantage of this technique is that it does not allow for longitudinal studies in individual animals.

## 3. Conclusion

In conclusion, there are several methods to evaluate regulation of retinal blood flow. For clinical application, dye-based angiography is often used to characterize features of the retinal vasculature, such as dye leakage. This method is very helpful for clinical monitoring in patients with diabetic retinopathy or retinal vein occlusion to evaluate ischemic fundus areas or dye leakage for initiation of further therapeutic steps. Other methods, such as OCTA have good depth resolution and allow for quantification of retinal blood flow by using bidirectional Doppler OCT. Due to the possibility of having precise anatomical information and measurement of retinal blood flow in the same data set, these methods can be helpful for follow up studies where morphological and quantitative data is required. However, these measurements are limited to larger vessels and studies in laboratory animals are limited. For metabolic imaging retinal oximetry represents a suitable method that is also limited to larger vessels and evaluation of capillary metabolism is not possible. To analyze retinal blood flow in laboratory animals, including gene-targeted mice, a higher spatial resolution is necessary. First-order arterioles in the mouse retina have typically luminal diameters between 20 and 30 μm. *Ex vivo* imaging of diameter changes by transmitted light microscopy showed excellent results with high optical resolution without the interference of blood pressure or anesthetics. Diameter changes of 1–3% are detectable with this method, depending on the vessel size. However, no longitudinal studies are possible with this technique and retinal preparation can be challenging for unexperienced investigators. Laser Speckle Flowgraphy represents a reliable method to evaluate total retinal blood flow or blood flow of an individual vessel *in vivo*, which enables longitudinal studies, also in small laboratory animals. However, *in vivo* studies in animals have the disadvantage that anesthetics or systemic blood pressure changes may influence retinal vascular responses, which is a drawback when local mechanisms of vessel diameter regulation are studied. Taken together, the individual methods for studying regulation of retinal blood flow and vessel diameter have their advantages and disadvantages. Hence, the best method for a specific experiment should be chosen depending on the objective of the study.

## Author contributions

EB, FW, and AG wrote the manuscript. NP made critical revisions. All authors contributed to the article and approved the submitted version.

## References

[1] Chua J Sim R Tan B Wong D Yao X Liu X Optical coherence tomography angiography in diabetes and diabetic retinopathy. *J Clin Med.* (2020) 9:1723. 10.3390/jcm9061723 32503234 PMC7357089

[2] Ejaz S Chekarova I Ejaz A Sohail A Lim C. Importance of pericytes and mechanisms of pericyte loss during diabetes retinopathy. *Diabetes Obes Metab.* (2008) 10:53–63. 10.1111/j.1463-1326.2007.00795.x 17941874

[3] Daruich A Matet A Moulin A Kowalczuk L Nicolas M Sellam A Mechanisms of macular edema: beyond the surface. *Prog Retin Eye Res.* (2018) 63:20–68. 10.1016/j.preteyeres.2017.10.006 29126927

[4] Pemp B Weigert G Karl K Petzl U Wolzt M Schmetterer L Correlation of flicker-induced and flow-mediated vasodilatation in patients with endothelial dysfunction and healthy volunteers. *Diabetes Care.* (2009) 32:1536–41. 10.2337/dc08-2130 19478197 PMC2713642

[5] Garhöfer G Kopf A Polska E Malec M Dorner G Wolzt M Influence of exercise induced hyperlactatemia on retinal blood flow during normo- and hyperglycemia. *Curr Eye Res.* (2004) 28:351–8. 10.1076/ceyr.28.5.351.28680 15287372

[6] Nguyen T Kawasaki R Wang J Kreis A Shaw J Vilser W Flicker light-induced retinal vasodilation in diabetes and diabetic retinopathy. *Diabetes Care.* (2009) 32:2075–80. 10.2337/dc09-0075 19641162 PMC2768208

[7] Scott I Campochiaro P Newman N Biousse V. Retinal vascular occlusions. *Lancet.* (2020) 396:1927–40. 10.1016/s0140-6736(20)31559-2 33308475 PMC9546635

[8] Kang M Balaratnasingam C Yu P Morgan W McAllister I Cringle S Alterations to vascular endothelium in the optic nerve head in patients with vascular comorbidities. *Exp Eye Res.* (2013) 111:50–60. 10.1016/j.exer.2013.03.005 23518407

[9] Janssen M den Heijer M Cruysberg J Wollersheim H Bredie S. Retinal vein occlusion: a form of venous thrombosis or a complication of atherosclerosis? A meta-analysis of thrombophilic factors. *Thromb Haemost.* (2005) 93:1021–6. 10.1160/th04-11-0768 15968383

[10] Jonas J Monés J Glacet-Bernard A Coscas G. Retinal vein occlusions. *Dev Ophthalmol.* (2017) 58:139–67. 10.1159/000455278 28351046

[11] Dominguez E Raoul W Calippe B Sahel J Guillonneau X Paques M Experimental branch retinal vein occlusion induces upstream pericyte loss and vascular destabilization. *PLoS One.* (2015) 10:e0132644. 10.1371/journal.pone.0132644 26208283 PMC4514656

[12] Biousse V Nahab F Newman N. Management of acute retinal ischemia: follow the guidelines! *Ophthalmology.* (2018) 125:1597–607. 10.1016/j.ophtha.2018.03.054 29716787

[13] Gericke A Mann C Zadeh J Musayeva A Wolff I Wang M Elevated intraocular pressure causes abnormal reactivity of mouse retinal arterioles. *Oxid Med Cell Longev.* (2019) 2019:9736047. 10.1155/2019/9736047 31976030 PMC6954472

[14] Wang M Liu H Xia N Li H van Beers T Gericke A Intraocular pressure-induced endothelial dysfunction of retinal blood vessels is persistent, but does not trigger retinal ganglion cell loss. *Antioxidants.* (2022) 11:1864. 10.3390/antiox11101864 36290587 PMC9598728

[15] Hou H Moghimi S Proudfoot J Ghahari E Penteado R Bowd C Ganglion cell complex thickness and macular vessel density loss in primary open-angle glaucoma. *Ophthalmology.* (2020) 127:1043–52. 10.1016/j.ophtha.2019.12.030 32085875 PMC7354893

[16] Cull G Burgoyne C Fortune B Wang L. Longitudinal hemodynamic changes within the optic nerve head in experimental glaucoma. *Invest Ophthalmol Vis Sci.* (2013) 54:4271–7. 10.1167/iovs.13-12013 23737471 PMC3691051

[17] Pennington K DeAngelis M. Epidemiology of age-related macular degeneration (AMD): associations with cardiovascular disease phenotypes and lipid factors. *Eye Vis.* (2016) 3:34. 10.1186/s40662-016-0063-5 28032115 PMC5178091

[18] Ruan Y Jiang S Gericke A. Age-related macular degeneration: role of oxidative stress and blood vessels. *Int Exp Ophthalmol.* (2021) 22:1296. 10.3390/ijms22031296 33525498 PMC7866075

[19] Yu D Cringle S. Retinal degeneration and local oxygen metabolism. *Exp Eye Res.* (2005) 80:745–51. 10.1016/j.exer.2005.01.018 15939030

[20] de Carlo T Romano A Waheed N Duker JS. A review of optical coherence tomography angiography (OCTA). *Int J Retina Vitreous.* (2015) 1:5. 10.1186/s40942-015-0005-8 27847598 PMC5066513

[21] Farazdaghi M Ebrahimi K. Role of the choroid in age-related macular degeneration: a current review. *J Ophthalmic Vis Res.* (2019) 14:78–87. 10.4103/jovr.jovr_125_18 30820291 PMC6388521

[22] Yeo N Chan E Cheung C. Choroidal neovascularization: mechanisms of endothelial dysfunction. *Front Pharmacol.* (2019) 10:1363. 10.3389/fphar.2019.01363 31849644 PMC6895252

[23] Yi X Ogata N Komada M Yamamoto C Takahashi K Omori K Vascular endothelial growth factor expression in choroidal neovascularization in rats. *Graefes Arch Clin Exp Ophthalmol.* (1997) 235:313–9. 10.1007/bf01739641 9176680

[24] Toulouie S Chang S Pan J Snyder K Yiu G. Relationship of retinal vessel caliber with age-related macular degeneration. *J Ophthalmol.* (2022) 2022:8210599. 10.1155/2022/8210599 35957743 PMC9357695

[25] Lee S Tran S Amin A Morse L Moshiri A Park S Retinal vessel density in exudative and nonexudative age-related macular degeneration on optical coherence tomography angiography. *Am J Ophthalmol.* (2020) 212:7–16. 10.1016/j.ajo.2019.11.031 31837316 PMC7113105

[26] Hartnett M Penn J. Mechanisms and management of retinopathy of prematurity. *N Engl J Med.* (2012) 367:2515–26. 10.1056/NEJMra1208129 23268666 PMC3695731

[27] Hartnett M. Pathophysiology and mechanisms of severe retinopathy of prematurity. *Ophthalmology.* (2015) 122:200–10. 10.1016/j.ophtha.2014.07.050 25444347 PMC4277936

[28] Tomita Y Usui-Ouchi A Nilsson A Yang J Ko M Hellström A Metabolism in retinopathy of prematurity. *Life.* (2021) 11:1119. 10.3390/life11111119 34832995 PMC8620873

[29] Bill A Sperber G. Control of retinal and choroidal blood flow. *Eye.* (1990) 4(Pt 2):319–25. 10.1038/eye.1990.43 2199239

[30] Furchgott R Zawadzki J. The obligatory role of endothelial cells in the relaxation of arterial smooth muscle by acetylcholine. *Nature.* (1980) 288:373–6. 10.1038/288373a0 6253831

[31] Sorrentino F Matteini S Bonifazzi C Sebastiani A Parmeggiani F. Diabetic retinopathy and endothelin system: microangiopathy versus endothelial dysfunction. *Eye.* (2018) 32:1157–63. 10.1038/s41433-018-0032-4 29520046 PMC6043602

[32] Ganesan P He S Xu H. Development of an image-based model for capillary vasculature of retina. *Comput Methods Prog Biomed.* (2011) 102:35–46. 10.1016/j.cmpb.2010.12.009 21277036

[33] Kornfield T Newman E. Regulation of blood flow in the retinal trilaminar vascular network. *J Neurosci.* (2014) 34:11504–13. 10.1523/jneurosci.1971-14.2014 25143628 PMC4138352

[34] Linsenmeier R. Effects of light and darkness on oxygen distribution and consumption in the cat retina. *J Gen Physiol.* (1986) 88:521–42. 10.1085/jgp.88.4.521 3783124 PMC2228847

[35] Riva C Logean E Falsini B. Visually evoked hemodynamical response and assessment of neurovascular coupling in the optic nerve and retina. *Prog Retin Eye Res.* (2005) 24:183–215. 10.1016/j.preteyeres.2004.07.002 15610973

[36] Polak K Dorner G Kiss B Polska E Findl O Rainer G Evaluation of the zeiss retinal vessel analyser. *Br J Ophthalmol.* (2000) 84:1285–90. 10.1136/bjo.84.11.1285 11049956 PMC1723319

[37] Seifertl B Vilser W. Retinal vessel analyzer (RVA)–design and function. *Biomed Eng.* (2002) 47 (Suppl. 1):678–81. 10.1515/bmte.2002.47.s1b.678 12465272

[38] Schmetterer L Garhofer G. How can blood flow be measured? *Surv Ophthalmol.* (2007) 52:S134–8. 10.1016/j.survophthal.2007.08.008 17998038

[39] Garhofer G Bek T Boehm A Gherghel D Grunwald J Jeppesen P Use of the retinal vessel analyzer in ocular blood flow research. *Acta Ophthalmol.* (2010) 88:717–22. 10.1111/j.1755-3768.2009.01587.x 19681764

[40] Polak K Schmetterer L Riva C. Influence of flicker frequency on flicker-induced changes of retinal vessel diameter. *Invest Ophthalmol Vis Sci.* (2002) 43:2721–6. 12147608

[41] Dorner G Garhöfer G Huemer K Riva C Wolzt M Schmetterer L. Hyperglycemia affects flicker-induced vasodilation in the retina of healthy subjects. *Vis Res.* (2003) 43:1495–500. 10.1016/s0042-6989(03)00170-6 12767316

[42] Garhöfer G Zawinka C Resch H Kothy P Schmetterer L Dorner G. Reduced response of retinal vessel diameters to flicker stimulation in patients with diabetes. *Br J Ophthalmol.* (2004) 88:887–91. 10.1136/bjo.2003.033548 15205231 PMC1772243

[43] Lim L Ling L Ong P Foulds W Tai E Wong T. Dynamic responses in retinal vessel caliber with flicker light stimulation and risk of diabetic retinopathy and its progression. *Invest Ophthalmol Vis Sci.* (2017) 58:2449–55. 10.1167/iovs.16-21008 28460046

[44] Kotliar K Lanzl I Schmidt-Trucksäss A Sitnikova D Ali M Blume K Dynamic retinal vessel response to flicker in obesity: a methodological approach. *Microvasc Res.* (2011) 81:123–8. 10.1016/j.mvr.2010.11.007 21094174

[45] Streese L Kotliar K Deiseroth A Infanger D Gugleta K Schmaderer C Retinal endothelial function in cardiovascular risk patients: a randomized controlled exercise trial. *Scand J Med Sci Sports.* (2020) 30:272–80. 10.1111/sms.13560 31580506

[46] Angermann S Günthner R Hanssen H Lorenz G Braunisch M Steubl D Cognitive impairment and microvascular function in end-stage renal disease. *Int J Methods Psychiatr Res.* (2022) 31:e1909. 10.1002/mpr.1909 35290686 PMC9159686

[47] Theuerle J Al-Fiadh A Amirul Islam F Patel S Burrell L Wong T Impaired retinal microvascular function predicts long-term adverse events in patients with cardiovascular disease. *Cardiovasc Res.* (2021) 117:1949–57. 10.1093/cvr/cvaa245 32750111

[48] Garhöfer G Zawinka C Resch H Huemer K Schmetterer L Dorner G. Response of retinal vessel diameters to flicker stimulation in patients with early open angle glaucoma. *J Glaucoma.* (2004) 13:340–4. 10.1097/00061198-200408000-00013 15226664

[49] Gugleta K Kochkorov A Waldmann N Polunina A Katamay R Flammer J Dynamics of retinal vessel response to flicker light in glaucoma patients and ocular hypertensives. *Graefes Arch Clin Exp Ophthalmol.* (2012) 250:589–94. 10.1007/s00417-011-1842-2 22008947

[50] Dorner G Garhofer G Kiss B Polska E Polak K Riva C Nitric oxide regulates retinal vascular tone in humans. *Am J Physiol Heart Circ Physiol.* (2003) 285:H631–6. 10.1152/ajpheart.00111.2003 12750062

[51] Lasta M Polak K Luksch A Garhofer G Schmetterer L. Effect of NO synthase inhibition on retinal vessel reaction to isometric exercise in healthy humans. *Acta Ophthalmol.* (2012) 90:362–8. 10.1111/j.1755-3768.2010.01970.x 20636485

[52] Albanna W Kotliar K Lüke J Alpdogan S Conzen C Lindauer U Non-invasive evaluation of neurovascular coupling in the murine retina by dynamic retinal vessel analysis. *PLoS One.* (2018) 13:e0204689. 10.1371/journal.pone.0204689 30286110 PMC6171857

[53] Neumaier F Kotliar K Haeren R Temel Y Lüke J Seyam O Retinal vessel responses to flicker stimulation are impaired in Ca (v) 2.3-deficient mice-an in-vivo evaluation using retinal vessel analysis (RVA). *Fronti Neurol.* (2021) 12:659890. 10.3389/fneur.2021.659890 33927686 PMC8076560

[54] Riva C Grunwald J Sinclair S O’Keefe K. Fundus camera based retinal LDV. *Appl Opt.* (1981) 20:117–20. 10.1364/ao.20.000117 20309075

[55] Riva C Feke G Eberli B Benary V. Bidirectional LDV system for absolute measurement of blood speed in retinal vessels. *Appl Opt.* (1979) 18:2301–6. 10.1364/ao.18.002301 20212650

[56] Riva C Grunwald J Sinclair S Petrig B. Blood velocity and volumetric flow rate in human retinal vessels. *Invest Ophthalmol Vis Sci.* (1985) 26:1124–32. 4019103

[57] Garhofer G Werkmeister R Dragostinoff N Schmetterer L. Retinal blood flow in healthy young subjects. *Invest Ophthalmol Vis Sci.* (2012) 53:698–703. 10.1167/iovs.11-8624 22247463

[58] Feke G Goger D Tagawa H Delori F. Laser Doppler technique for absolute measurement of blood speed in retinal vessels. *IEEE Trans Bio Med Eng.* (1987) 34:673–80. 10.1109/tbme.1987.325992 2958402

[59] Guan K Hudson C Flanagan J. Variability and repeatability of retinal blood flow measurements using the canon laser blood flowmeter. *Microvasc Res.* (2003) 65:145–51. 10.1016/s0026-2862(03)00007-4 12711255

[60] Garhöfer G Zawinka C Resch H Huemer K Dorner G Schmetterer L. Diffuse luminance flicker increases blood flow in major retinal arteries and veins. *Vis Res.* (2004) 44:833–8. 10.1016/j.visres.2003.11.013 14967208

[61] Garhöfer G Resch H Sacu S Weigert G Schmidl D Lasta M Effect of regular smoking on flicker induced retinal vasodilatation in healthy subjects. *Microvasc Res.* (2011) 82:351–5. 10.1016/j.mvr.2011.07.001 21771603

[62] Bonner R Nossal R. Principles of laser-doppler flowmetry. In: Shepherd A Öberg P editors. *Laser-Doppler Blood Flowmetry.* Boston, MA: Springer US (1990). p. 17–45.

[63] Riva C Cranstoun S Grunwald J Petrig B. Choroidal blood flow in the foveal region of the human ocular fundus. *Invest Ophthalmol Vis Sci.* (1994) 35:4273–81. 8002247

[64] Garhöfer G Resch H Weigert G Lung S Simader C Schmetterer L. Short-term increase of intraocular pressure does not alter the response of retinal and optic nerve head blood flow to flicker stimulation. *Invest ophthalmol Vis Sci.* (2005) 46:1721–5. 10.1167/iovs.04-1347 15851574

[65] Bata A Fondi K Witkowska K Werkmeister R Hommer A Vass C Optic nerve head blood flow regulation during changes in arterial blood pressure in patients with primary open-angle glaucoma. *Acta ophthalmol.* (2019) 97:e36–41. 10.1111/aos.13850 30218499 PMC6492118

[66] Boltz A Told R Napora K Palkovits S Werkmeister R Schmidl D Optic nerve head blood flow autoregulation during changes in arterial blood pressure in healthy young subjects. *PLoS One.* (2013) 8:e82351. 10.1371/journal.pone.0082351 24324774 PMC3855769

[67] Polska E Polak K Luksch A Fuchsjager-Mayrl G Petternel V Findl O Twelve hour reproducibility of choroidal blood flow parameters in healthy subjects. *Br J Ophthalmol.* (2004) 88:533–7. 10.1136/bjo.2003.028480 15031172 PMC1772102

[68] Strenn K Menapace R Rainer G Findl O Wolzt M Schmetterer L. Reproducibility and sensitivity of scanning laser Doppler flowmetry during graded changes in PO2. *Br J Ophthalmol.* (1997) 81:360–4. 10.1136/bjo.81.5.360 9227199 PMC1722185

[69] Michelson G Schmauss B Langhans M Harazny J Groh M. Principle, validity, and reliability of scanning laser Doppler flowmetry. *J Glaucoma.* (1996) 5:99–105. 8795741

[70] Chauhan B Yu P Cringle S Yu D. Confocal scanning laser Doppler flowmetry in the rat retina: origin of flow signals and dependence on scan depth. *Arch Ophthalmol.* (2006) 124:397–402. 10.1001/archopht.124.3.397 16534060

[71] Wolf S Jung F Kiesewetter H Körber N Reim M. Video fluorescein angiography: method and clinical application. *Graefes Arch Clin Exp Ophthalmol.* (1989) 227:145–51. 10.1007/bf02169788 2721984

[72] Weinhaus R Burke J Delori F Snodderly D. Comparison of fluorescein angiography with microvascular anatomy of macaque retinas. *Exp Eye Res.* (1995) 61:1–16. 10.1016/s0014-4835(95)80053-0 7556462

[73] Spaide R Fujimoto J Waheed N Sadda S Staurenghi G. Optical coherence tomography angiography. *Prog Retin Eye Res.* (2018) 64:1–55. 10.1016/j.preteyeres.2017.11.003 29229445 PMC6404988

[74] Chui T Dubow M Pinhas A Shah N Gan A Weitz R Comparison of adaptive optics scanning light ophthalmoscopic fluorescein angiography and offset pinhole imaging. *Biomed Opt Express.* (2014) 5:1173–89. 10.1364/boe.5.001173 24761299 PMC3985984

[75] Kwan A Barry C McAllister I Constable I. Fluorescein angiography and adverse drug reactions revisited: the Lions Eye experience. *Clin Exp Ophthalmol.* (2006) 34:33–8. 10.1111/j.1442-9071.2006.01136.x 16451256

[76] Kwiterovich K Maguire M Murphy R Schachat A Bressler N Bressler S Frequency of adverse systemic reactions after fluorescein angiography. Results of a prospective study. *Ophthalmology.* (1991) 98:1139–42. 10.1016/s0161-6420(91)32165-1 1891225

[77] de Carlo T Bonini Filho M Baumal C Reichel E Rogers A Witkin A Evaluation of preretinal neovascularization in proliferative diabetic retinopathy using optical coherence tomography angiography. *Ophthalmic Surg Lasers Imaging Retina.* (2016) 47:115–9. 10.3928/23258160-20160126-03 26878443

[78] Matsunaga D Yi J De Koo L Ameri H Puliafito C Kashani A. Optical coherence tomography angiography of diabetic retinopathy in human subjects. *Ophthalmic Surg Lasers Imaging Retina.* (2015) 46:796–805. 10.3928/23258160-20150909-03 26431294

[79] Kim A Chu Z Shahidzadeh A Wang R Puliafito C Kashani A. Quantifying microvascular density and morphology in diabetic retinopathy using spectral-domain optical coherence tomography angiography. *Invest Ophthalmol Vis Sci.* (2016) 57:362–70. 10.1167/iovs.15-18904 27409494 PMC4968771

[80] Nesper P Roberts P Onishi A Chai H Liu L Jampol L Quantifying microvascular abnormalities with increasing severity of diabetic retinopathy using optical coherence tomography angiography. *Invest Ophthalmol Vis Sci.* (2017) 58:307–15. 10.1167/iovs.17-21787 29059262 PMC5693005

[81] Hirano T Kitahara J Toriyama Y Kasamatsu H Murata T Sadda S. Quantifying vascular density and morphology using different swept-source optical coherence tomography angiographic scan patterns in diabetic retinopathy. *Br J Ophthalmol.* (2019) 103:216–21. 10.1136/bjophthalmol-2018-311942 29706601

[82] Lu Y Simonett J Wang J Zhang M Hwang T Hagag A Evaluation of automatically quantified foveal avascular zone metrics for diagnosis of diabetic retinopathy using optical coherence tomography angiography. *Invest Ophthalmol Vis Sci.* (2018) 59:2212–21. 10.1167/iovs.17-23498 29715365 PMC5958306

[83] Lei J Yi E Suo Y Chen C Xu X Ding W Distinctive analysis of macular superficial capillaries and large vessels using optical coherence tomographic angiography in healthy and diabetic eyes. *Invest Ophthalmol Vis Sci.* (2018) 59:1937–43. 10.1167/iovs.17-23676 29677360

[84] Sousa D Leal I Moreira S do Vale S Silva-Herdade A Aguiar P A protocol to evaluate retinal vascular response using optical coherence tomography angiography. *Front Neurosci.* (2019) 13:566. 10.3389/fnins.2019.00566 31249500 PMC6582622

[85] Nesper P Lee H Fayed A Schwartz G Yu F Fawzi A. Hemodynamic response of the three macular capillary plexuses in dark adaptation and flicker stimulation using optical coherence tomography angiography. *Invest Ophthalmol Vis Sci.* (2019) 60:694–703. 10.1167/iovs.18-25478 30786274 PMC6383834

[86] Rao H Pradhan Z Suh M Moghimi S Mansouri K Weinreb R. Optical coherence tomography angiography in glaucoma. *J Glaucoma.* (2020) 29:312–21. 10.1097/ijg.0000000000001463 32053551 PMC7117982

[87] Rao H Pradhan Z Weinreb R Reddy H Riyazuddin M Dasari S Regional comparisons of optical coherence tomography angiography vessel density in primary open-angle glaucoma. *Am J Ophthalmol.* (2016) 171:75–83. 10.1016/j.ajo.2016.08.030 27590118

[88] Takusagawa H Liu L Ma K Jia Y Gao S Zhang M Projection-resolved optical coherence tomography angiography of macular retinal circulation in glaucoma. *Ophthalmology.* (2017) 124:1589–99. 10.1016/j.ophtha.2017.06.002 28676279 PMC5651191

[89] Shin J Lee J Kwon J Choi J Kook M. Regional vascular density-visual field sensitivity relationship in glaucoma according to disease severity. *Br J Ophthalmol.* (2017) 101:1666–72. 10.1136/bjophthalmol-2017-310180 28432111

[90] Ma Z Pan X Zhou D Zhu Z Xu A Shi P Changes of retinal and choroidal capillary blood flow in macula after an acute intraocular pressure elevation. *Medicine.* (2020) 99:e21007. 10.1097/md.0000000000021007 32590817 PMC7328993

[91] Alnawaiseh M Rosentreter A Hillmann A Alex A Niekämper D Heiduschka P OCT angiography in the mouse: a novel evaluation method for vascular pathologies of the mouse retina. *Exp Eye Res.* (2016) 145:417–23. 10.1016/j.exer.2016.02.012 26946073

[92] Alnawaiseh M Brand C Bormann E Wistuba J Eter N Heiduschka P. Quantitative analysis of retinal perfusion in mice using optical coherence tomography angiography. *Exp Eye Res.* (2017) 164:151–6. 10.1016/j.exer.2017.09.003 28889963

[93] Uehara H Lesuma T Stocking P Jensen N Kumar S Zhang M Detection of microvascular retinal changes in type I diabetic mice with optical coherence tomography angiography. *Exp Eye Res.* (2019) 178:91–8. 10.1016/j.exer.2018.09.017 30268699 PMC6361705

[94] Giannakaki-Zimmermann H Kokona D Wolf S Ebneter A Zinkernagel M. Optical coherence tomography angiography in mice: comparison with confocal scanning laser microscopy and fluorescein angiography. *Transl Vis Sci Technol.* (2016) 5:11. 10.1167/tvst.5.4.11 27570710 PMC4997887

[95] Spaide R Fujimoto J Waheed N. Image artifacts in optical coherence tomography angiography. *Retina.* (2015) 35:2163–80. 10.1097/iae.0000000000000765 26428607 PMC4712934

[96] Cole ED Moult E Dang S Choi W Ploner S Lee B The definition, rationale, and effects of thresholding in OCT angiography. *Ophthalmol Retina.* (2017) 1:435–47. 10.1016/j.oret.2017.01.019 29034359 PMC5640169

[97] La Mantia A Kurt R Mejor S Egan C Tufail A Keane P Comparing fundus fluorescein angiography and swept-source optical coherence tomography angiography in the evaluation of diabetic macular perfusion. *Retina.* (2019) 39:926–37. 10.1097/iae.0000000000002045 29346244

[98] Couturier A Mané V Bonnin S Erginay A Massin P Gaudric A Capillary plexus anomalies in diabetic retinopathy on optical coherence tomography angiography. *Retina.* (2015) 35:2384–91. 10.1097/iae.0000000000000859 26469531

[99] Salz D de Carlo T Adhi M Moult E Choi W Baumal C Select features of diabetic retinopathy on swept-source optical coherence tomographic angiography compared with fluorescein angiography and normal eyes. *JAMA Ophthalmol.* (2016) 134:644–50. 10.1001/jamaophthalmol.2016.0600 27055248 PMC5312730

[100] Yao X Ke M Ho Y Lin E Wong D Tan B Comparison of retinal vessel diameter measurements from swept-source OCT angiography and adaptive optics ophthalmoscope. *Br J Ophthalmol.* (2021) 105:426–31. 10.1136/bjophthalmol-2020-316111 32461263 PMC7907556

[101] Ghasemi Falavarjani K Al-Sheikh M Darvizeh F Sadun A Sadda S. Retinal vessel calibre measurements by optical coherence tomography angiography. *Br J Ophthalmol.* (2017) 101:989–92. 10.1136/bjophthalmol-2016-309678 27852583

[102] Kim S Kong R Popel A Intaglietta M Johnson PC. A computer-based method for determination of the cell-free layer width in microcirculation. *Microcirculation.* (2006) 13:199–207. 10.1080/10739680600556878 16627362

[103] Yu P Mehnert A Athwal A Sarunic M Yu D. Use of the retinal vascular histology to validate an optical coherence tomography angiography technique. *Transl Vis Sci Technol.* (2021) 10:29. 10.1167/tvst.10.1.29 33520424 PMC7817878

[104] de Carlo T Chin A Bonini Filho M Adhi M Branchini L Salz D Detection Of microvascular changes in eyes of patients with diabetes but not clinical diabetic retinopathy using optical coherence tomography angiography. *Retina.* (2015) 35:2364–70. 10.1097/iae.0000000000000882 26469537

[105] Krawitz B Mo S Geyman L Agemy S Scripsema N Garcia P Acircularity index and axis ratio of the foveal avascular zone in diabetic eyes and healthy controls measured by optical coherence tomography angiography. *Vis Res.* (2017) 139:177–86. 10.1016/j.visres.2016.09.019 28212983

[106] Tang F Ng D Lam A Luk F Wong R Chan C Determinants of quantitative optical coherence tomography angiography metrics in patients with diabetes. *Sci Rep.* (2017) 7:2575. 10.1038/s41598-017-02767-0 28566760 PMC5451475

[107] Freiberg F Pfau M Wons J Wirth M Becker M Michels S. Optical coherence tomography angiography of the foveal avascular zone in diabetic retinopathy. *Graefes Arch Clin Exp Ophthalmol.* (2016) 254:1051–8. 10.1007/s00417-015-3148-2 26338819 PMC4884570

[108] Shahlaee A Pefkianaki M Hsu J Ho A. Measurement of foveal avascular zone dimensions and its reliability in healthy eyes using optical coherence tomography angiography. *Am J Ophthalmol.* (2016) 161:50–5.e1. 10.1016/j.ajo.2015.09.026 26423672

[109] Lupidi M Coscas F Cagini C Fiore T Spaccini E Fruttini D Automated quantitative analysis of retinal microvasculature in normal eyes on optical coherence tomography angiography. *Am J Ophthalmol.* (2016) 169:9–23. 10.1016/j.ajo.2016.06.008 27296485

[110] Coscas F Sellam A Glacet-Bernard A Jung C Goudot M Miere A Normative data for vascular density in superficial and deep capillary plexuses of healthy adults assessed by optical coherence tomography angiography. *Invest Ophthalmol Vis Sci.* (2016) 57:211–23. 10.1167/iovs.15-18793 27409475

[111] Carpineto P Mastropasqua R Marchini G Toto L Di Nicola M Di Antonio L. Reproducibility and repeatability of foveal avascular zone measurements in healthy subjects by optical coherence tomography angiography. *Br J Ophthalmol.* (2016) 100:671–6. 10.1136/bjophthalmol-2015-307330 26377414

[112] La Spina C Carnevali A Marchese A Querques G Bandello F. Reproducibility and reliability of optical coherence tomography angiography for foveal avascular zone evaluation and measurement in different settings. *Retina.* (2017) 37:1636–41. 10.1097/iae.0000000000001426 28002271

[113] Linderman R Salmon A Strampe M Russillo M Khan J Carroll J. Assessing the accuracy of foveal avascular zone measurements using optical coherence tomography angiography: segmentation and scaling. *Transl Vis Sci Technol.* (2017) 6:16. 10.1167/tvst.6.3.16 28616362 PMC5469394

[114] Li C Lee C. Minimum cross entropy thresholding. *Pattern Recognit.* (1993) 26:617–25. 10.1016/0031-3203(93)90115-D

[115] Leitgeb R Werkmeister R Blatter C Schmetterer L. Doppler optical coherence tomography. *Prog Retin Eye Res.* (2014) 41:26–43. 10.1016/j.preteyeres.2014.03.004 24704352 PMC4073226

[116] Werkmeister R Dragostinoff N Palkovits S Told R Boltz A Leitgeb R Measurement of absolute blood flow velocity and blood flow in the human retina by dual-beam bidirectional Doppler fourier-domain optical coherence tomography. *Invest Ophthalmol Vis Sci.* (2012) 53:6062–71. 10.1167/iovs.12-9514 22893675

[117] Wang Y Bower B Izatt J Tan O Huang D. In vivo total retinal blood flow measurement by Fourier domain Doppler optical coherence tomography. *J Biomed Opt.* (2007) 12:041215. 10.1117/1.2772871 17867804

[118] Singh A Kolbitsch C Schmoll T Leitgeb R. Stable absolute flow estimation with Doppler OCT based on virtual circumpapillary scans. *Biomed Opt Express.* (2010) 1:1047–58. 10.1364/boe.1.001047 21258529 PMC3018083

[119] Wang Y Lu A Gil-Flamer J Tan O Izatt J Huang D. Measurement of total blood flow in the normal human retina using Doppler Fourier-domain optical coherence tomography. *Br J Ophthalmol.* (2009) 93:634–7. 10.1136/bjo.2008.150276 19168468 PMC2743389

[120] Davé D Milner T. Doppler-angle measurement in highly scattering media. *Opt Lett.* (2000) 25:1523–5. 10.1364/ol.25.001523 18066266

[121] Werkmeister R Dragostinoff N Pircher M Götzinger E Hitzenberger C Leitgeb R Bidirectional Doppler fourier-domain optical coherence tomography for measurement of absolute flow velocities in human retinal vessels. *Opt Lett.* (2008) 33:2967–9. 10.1364/ol.33.002967 19079508

[122] Szegedi S Hommer N Kallab M Puchner S Schmidl D Werkmeister R Repeatability and reproducibility of total retinal blood flow measurements using bi-directional Doppler OCT. *Transl Vis Sci Technol.* (2020) 9:34. 10.1167/tvst.9.7.34 32832239 PMC7414639

[123] Wang Y Fawzi A Tan O Gil-Flamer J Huang D. Retinal blood flow detection in diabetic patients by Doppler Fourier domain optical coherence tomography. *Opt Express.* (2009) 17:4061–73. 10.1364/oe.17.004061 19259246 PMC2821425

[124] Wang Y Fawzi A Varma R Sadun A Zhang X Tan O Pilot study of optical coherence tomography measurement of retinal blood flow in retinal and optic nerve diseases. *Invest Ophthalmol Vis Sci.* (2011) 52:840–5. 10.1167/iovs.10-5985 21051715 PMC3053109

[125] Wang Y Fawzi A Tan O Zhang X Huang D. Flicker-induced changes in retinal blood flow assessed by Doppler optical coherence tomography. *Biomed Opt Express.* (2011) 2:1852–60. 10.1364/boe.001852 21750763 PMC3130572

[126] Aschinger G Schmetterer L Fondi K Aranha Dos Santos V Seidel G Garhöfer G Effect of diffuse luminance flicker light stimulation on total retinal blood flow assessed with dual-beam bidirectional Doppler OCT. *Invest Ophthalmol Vis Sci.* (2017) 58:1167–78. 10.1167/iovs.16-20598 28245297

[127] Werkmeister R Schmidl D Aschinger G Doblhoff-Dier V Palkovits S Wirth M Retinal oxygen extraction in humans. *Sci Rep.* (2015) 5:15763. 10.1038/srep15763 26503332 PMC4621499

[128] Doblhoff-Dier V Schmetterer L Vilser W Garhöfer G Gröschl M Leitgeb R Measurement of the total retinal blood flow using dual beam Fourier-domain Doppler optical coherence tomography with orthogonal detection planes. *Biomed Opt Express.* (2014) 5:630–42. 10.1364/boe.5.000630 24575355 PMC3920891

[129] Fondi K Aschinger G Bata A Wozniak P Liao L Seidel G Measurement of retinal vascular caliber from optical coherence tomography phase images. *Invest Ophthalmol Vis Sci.* (2016) 57:121–9. 10.1167/iovs.15-18476 27409462

[130] Stefánsson E Olafsdottir O Eliasdottir T Vehmeijer W Einarsdottir A Bek T Retinal oximetry: metabolic imaging for diseases of the retina and brain. *Prog Retin Eye Res.* (2019) 70:1–22. 10.1016/j.preteyeres.2019.04.001 30999027

[131] Beach J Schwenzer K Srinivas S Kim D Tiedeman J. Oximetry of retinal vessels by dual-wavelength imaging: calibration and influence of pigmentation. *J Appl Physiol.* (1999) 86:748–58. 10.1152/jappl.1999.86.2.748 9931217

[132] Schweitzer D Hammer M Kraft J Thamm E Königsdörffer E Strobel J. In vivo measurement of the oxygen saturation of retinal vessels in healthy volunteers. *IEEE Trans Bio Med Eng.* (1999) 46:1454–65. 10.1109/10.804573 10612903

[133] Geirsdottir A Palsson O Hardarson S Olafsdottir O Kristjansdottir J Stefánsson E. Retinal vessel oxygen saturation in healthy individuals. *Invest Ophthalmol Vis Sci.* (2012) 53:5433–42. 10.1167/iovs.12-9912 22786895

[134] Hammer M Vilser W Riemer T Schweitzer D. Retinal vessel oximetry-calibration, compensation for vessel diameter and fundus pigmentation, and reproducibility. *J Biomed Opt.* (2008) 13:054015. 10.1117/1.2976032 19021395

[135] Told R Boltz A Schmetterer L Garhöfer G Sacu S Schmidt-Erfurth U Method comparison of two non-invasive dual-wavelength spectrophotometric retinal oximeters in healthy young subjects during normoxia. *Acta Ophthalmol.* (2018) 96:e614–8. 10.1111/aos.13719 29488329

[136] Liu X Wang S Liu Y Liu L Lv Y Tang P Retinal oxygen saturation in Chinese adolescents. *Acta Ophthalmol.* (2017) 95:e54–61. 10.1111/aos.13167 27807947

[137] Nakano Y Shimazaki T Kobayashi N Miyoshi Y Ono A Kobayashi M Retinal oximetry in a healthy Japanese population. *PLoS One.* (2016) 11:e0159650. 10.1371/journal.pone.0159650 27434373 PMC4951009

[138] Palsson O Geirsdottir A Hardarson S Olafsdottir O Kristjansdottir J Stefánsson E. Retinal oximetry images must be standardized: a methodological analysis. *Invest Ophthalmol Vis Sci.* (2012) 53:1729–33. 10.1167/iovs.11-8621 22395877

[139] Jani P Mwanza J Billow K Waters A Moyer S Garg S. Normative values and predictors of retinal oxygen saturation. *Retina.* (2014) 34:394–401. 10.1097/IAE.0b013e3182979e7b 23842102

[140] Man R Sasongko M Kawasaki R Noonan J Lo T Luu C Associations of retinal oximetry in healthy young adults. *Invest Ophthalmol Vis Sci.* (2014) 55:1763–9. 10.1167/iovs.13-13320 24526435

[141] Zheng Q Zong Y Li L Huang X Lin L Yang W Retinal vessel oxygen saturation and vessel diameter in high myopia. *Ophthalmic Physiol Opt.* (2015) 35:562–9. 10.1111/opo.12223 26303449

[142] Olafsdottir O Eliasdottir T Kristjansdottir J Hardarson S Stefánsson E. Retinal Vessel oxygen saturation during 100% oxygen breathing in healthy individuals. *PLoS One.* (2015) 10:e0128780. 10.1371/journal.pone.0128780 26042732 PMC4456093

[143] Hickam J Sieker H Frayser R. Studies of retinal circulation and A-V oxygen difference in man. *Trans Am Clin Climatol Assoc.* (1959) 71:34–44. 14401681 PMC2248999

[144] Delaey C Boussery K Van de Voorde J. A retinal-derived relaxing factor mediates the hypoxic vasodilation of retinal arteries. *Invest Ophthalmol Vis Sci.* (2000) 41:3555–60. 11006252

[145] Hardarson S Basit S Jonsdottir T Eysteinsson T Halldorsson G Karlsson R Oxygen saturation in human retinal vessels is higher in dark than in light. *Invest Ophthalmol Vis Sci.* (2009) 50:2308–11. 10.1167/iovs.08-2576 19117923

[146] Hammer M Vilser W Riemer T Liemt F Jentsch S Dawczynski J Retinal venous oxygen saturation increases by flicker light stimulation. *Invest Ophthalmol Vis Sci.* (2011) 52:274–7. 10.1167/iovs.10-5537 20671271

[147] Palkovits S Lasta M Told R Schmidl D Werkmeister R Cherecheanu A Relation of retinal blood flow and retinal oxygen extraction during stimulation with diffuse luminance flicker. *Sci Rep.* (2015) 5:18291. 10.1038/srep18291 26672758 PMC4682144

[148] Palkovits S Lasta M Told R Schmidl D Boltz A Napora K Retinal oxygen metabolism during normoxia and hyperoxia in healthy subjects. *Invest Ophthalmol Vis Sci.* (2014) 55:4707–13. 10.1167/iovs.14-14593 25015353

[149] Palkovits S Told R Schmidl D Boltz A Napora K Lasta M Regulation of retinal oxygen metabolism in humans during graded hypoxia. *Am J Physiol Heart Circ Physiol.* (2014) 307:H1412–8. 10.1152/ajpheart.00479.2014 25217648

[150] Linsenmeier R Zhang H. Retinal oxygen: from animals to humans. *Prog Retin Eye Res.* (2017) 58:115–51. 10.1016/j.preteyeres.2017.01.003 28109737 PMC5441959

[151] Tamplin M Broadhurst K Vitale A Hashimoto R Kardon R Grumbach I. Longitudinal testing of retinal blood flow in a mouse model of hypertension by laser speckle flowgraphy. *Transl Vis Sci Technol.* (2021) 10:16. 10.1167/tvst.10.2.16 34003901 PMC7884297

[152] Wang L Cull G Piper C Burgoyne C Fortune B. Anterior and posterior optic nerve head blood flow in nonhuman primate experimental glaucoma model measured by laser speckle imaging technique and microsphere method. *Invest Ophthalmol Vis Sci.* (2012) 53:8303–9. 10.1167/iovs.12-10911 23169886 PMC3525139

[153] Sugiyama T Araie M Riva C Schmetterer L Orgul S. Use of laser speckle flowgraphy in ocular blood flow research. *Acta Ophthalmol.* (2010) 88:723–9. 10.1111/j.1755-3768.2009.01586.x 19725814

[154] Anraku A Enomoto N Tomita G Iwase A Sato T Shoji N Ocular and systemic factors affecting laser speckle flowgraphy measurements in the optic nerve head. *Transl Vis Sci Technol.* (2021) 10:13. 10.1167/tvst.10.1.13 33510952 PMC7804520

[155] Fukami M Iwase T Yamamoto K Kaneko H Yasuda S Terasaki H. Changes in retinal microcirculation after intravitreal ranibizumab injection in eyes with macular edema secondary to branch retinal vein occlusion. *Invest Ophthalmol Vis Sci.* (2017) 58:1246–55. 10.1167/iovs.16-21115 28241312

[156] Fondi K Bata A Luft N Witkowska K Werkmeister R Schmidl D Evaluation of flicker induced hyperemia in the retina and optic nerve head measured by laser speckle flowgraphy. *PLoS One.* (2018) 13:e0207525. 10.1371/journal.pone.0207525 30485331 PMC6261588

[157] Luft N Wozniak P Aschinger G Fondi K Bata A Werkmeister R Measurements of retinal perfusion using laser speckle flowgraphy and doppler optical coherence tomography. *Invest Ophthalmol Vis Sci.* (2016) 57:5417–25. 10.1167/iovs.16-19896 27756076

[158] Sato T Sugawara J Aizawa N Iwama N Takahashi F Nakamura-Kurakata M Longitudinal changes of ocular blood flow using laser speckle flowgraphy during normal pregnancy. *PLoS One.* (2017) 12:e0173127. 10.1371/journal.pone.0173127 28257508 PMC5336228

[159] Aizawa N Yokoyama Y Chiba N Omodaka K Yasuda M Otomo T Reproducibility of retinal circulation measurements obtained using laser speckle flowgraphy-NAVI in patients with glaucoma. *Clin Ophthalmol.* (2011) 5:1171–6. 10.2147/opth.s22093 21887100 PMC3162298

[160] Enaida H Okamoto K Fujii H Ishibashi T. LSFG findings of proliferative diabetic retinopathy after intravitreal injection of bevacizumab. *Ophthalmic Surg Lasers Imaging.* (2010) 41:e1–3. 10.3928/15428877-20101124-11 21117574

[161] Hashimoto R Sugiyama T Ubuka M Maeno T. Impairment of autoregulation of optic nerve head blood flow during vitreous surgery in patients with hypertension and hyperlipidemia. *Graefes Arch Clin Exp Ophthalmol.* (2017) 255:2227–35. 10.1007/s00417-017-3788-5 28940022

[162] Wada Y Higashide T Nagata A Sugiyama K. Longitudinal changes in optic nerve head blood flow in normal rats evaluated by laser speckle flowgraphy. *Invest Ophthalmol Vis Sci.* (2016) 57:5568–75. 10.1167/iovs.16-19945 27768795

[163] Takako H Hideki C Nobuhisa N. Evaluation of optic nerve head blood flow in normal rats and a rodent model of non-arteritic ischemic optic neuropathy using laser speckle flowgraphy. *Graefes Arch Clin Exp Ophthalmol.* (2017) 255:1973–80. 10.1007/s00417-017-3753-3 28786024

[164] Kida T Flammer J Oku H Konieczka K Morishita S Horie T Vasoactivity of retinal veins: a potential involvement of endothelin-1 (ET-1) in the pathogenesis of retinal vein occlusion (RVO). *Exp Eye Res.* (2018) 176:207–9. 10.1016/j.exer.2018.07.016 30025919

[165] Chaurasia S Lim R Parikh B Wey Y Tun B Wong T The NLRP3 inflammasome may contribute to pathologic neovascularization in the advanced stages of diabetic retinopathy. *Sci Rep.* (2018) 8:2847. 10.1038/s41598-018-21198-z 29434227 PMC5809448

[166] Hanaguri J Yokota H Watanabe M Kuo L Yamagami S Nagaoka T. Longitudinal stability of retinal blood flow regulation in response to flicker stimulation and systemic hyperoxia in mice assessed with laser speckle flowgraphy. *Sci Rep.* (2020) 10:19796. 10.1038/s41598-020-75296-y 33188259 PMC7666208

[167] Tamplin M Broadhurst K Vitale A Hashimoto R Kardon R Grumbach I. Measuring hyperemic response to light flicker stimulus using continuous laser speckle flowgraphy in mice. *Exp Eye Res.* (2022) 216:108952. 10.1016/j.exer.2022.108952 35051429 PMC9014798

[168] Nishinaka A Fuma S Inoue Y Shimazawa M Hara H. Effects of kallidinogenase on retinal edema and size of non-perfused areas in mice with retinal vein occlusion. *J Pharmacol Sci.* (2017) 134:86–92. 10.1016/j.jphs.2017.05.003 28619445

[169] Aizawa N Kunikata H Nitta F Shiga Y Omodaka K Tsuda S Age-and sex-dependency of laser speckle flowgraphy measurements of optic nerve vessel microcirculation. *PLoS One.* (2016) 11:e0148812. 10.1371/journal.pone.0148812 26872348 PMC4752292

[170] Moult E Choi W Boas D Baumann B Clermont A Feener E Evaluating anesthetic protocols for functional blood flow imaging in the rat eye. *J Biomed Opt.* (2017) 22:16005. 10.1117/1.jbo.22.1.016005 PMC5217081 28056146

[171] Kornfield T Newman E. Measurement of retinal blood flow using fluorescently labeled red blood cells. *Eneuro.* (2015) 2:ENEURO.0005-15.2015. 10.1523/eneuro.0005-15.2015 26082942 PMC4465795

[172] Biesecker K Srienc A Shimoda A Agarwal A Bergles D Kofuji P Glial cell calcium signaling mediates capillary regulation of blood flow in the retina. *J Neurosci.* (2016) 36:9435–45. 10.1523/jneurosci.1782-16.2016 27605617 PMC5013190

[173] Tadayoni R Paques M Gaudric A Vicaut E. Erythrocyte and leukocyte dynamics in the retinal capillaries of diabetic mice. *Exp Eye Res.* (2003) 77:497–504. 10.1016/s0014-4835(03)00155-6 12957148

[174] Wajer S Taomoto M McLeod D McCally R Nishiwaki H Fabry M Velocity measurements of normal and sickle red blood cells in the rat retinal and choroidal vasculatures. *Microvasc Res.* (2000) 60:281–93. 10.1006/mvre.2000.2270 11078644

[175] Jeon J Hwang Y Lee J Kong E Moon J Hong S Intravital imaging of circulating red blood cells in the retinal vasculature of growing mice. *Transl Vis Sci Technol.* (2021) 10:31. 10.1167/tvst.10.4.31 34004010 PMC8083064

[176] Lorentz K Zayas-Santiago A Tummala S Kang Derwent J. Scanning laser ophthalmoscope-particle tracking method to assess blood velocity during hypoxia and hyperoxia. *Adv Exp Med Biol.* (2008) 614:253–61. 10.1007/978-0-387-74911-2_29 18290336

[177] Ahmed J Pulfer M Linsenmeier R. Measurement of blood flow through the retinal circulation of the cat during normoxia and hypoxemia using fluorescent microspheres. *Microvasc Res.* (2001) 62:143–53. 10.1006/mvre.2001.2321 11516243

[178] Leskova W Watts M Carter P Eshaq R Harris N. Measurement of retinal blood flow rate in diabetic rats: disparity between techniques due to redistribution of flow. *Invest Ophthalmol Vis Sci.* (2013) 54:2992–9. 10.1167/iovs.13-11915 23572104 PMC3638664

[179] Werkmeister R Vietauer M Knopf C Fürnsinn C Leitgeb R Reitsamer H Measurement of retinal blood flow in the rat by combining Doppler Fourier-domain optical coherence tomography with fundus imaging. *J Biomed Opt.* (2014) 19:106008. 10.1117/1.jbo.19.10.106008 25321400

[180] Franceschini M Radhakrishnan H Thakur K Wu W Ruvinskaya S Carp S The effect of different anesthetics on neurovascular coupling. *Neuroimage.* (2010) 51:1367–77. 10.1016/j.neuroimage.2010.03.060 20350606 PMC2879067

[181] Ganesan P He S Xu H. Analysis of retinal circulation using an image-based network model of retinal vasculature. *Microvasc Res.* (2010) 80:99–109. 10.1016/j.mvr.2010.02.005 20156460

[182] Gericke A Goloborodko E Pfeiffer N Manicam C. Preparation steps for measurement of reactivity in mouse retinal arterioles ex vivo. *J Vis Exp.* (2018) 135:56199. 10.3791/56199 29806833 PMC6101129

[183] Böhmer T Manicam C Steege A Michel M Pfeiffer N Gericke A. The α_1_B -adrenoceptor subtype mediates adrenergic vasoconstriction in mouse retinal arterioles with damaged endothelium. *Br J Pharmacol.* (2014) 171:3858–67. 10.1111/bph.12743 24749494 PMC4128048

[184] Gericke A Sniatecki J Goloborodko E Steege A Zavaritskaya O Vetter J Identification of the muscarinic acetylcholine receptor subtype mediating cholinergic vasodilation in murine retinal arterioles. *Invest Ophthalmol Vis Sci.* (2011) 52:7479–84. 10.1167/iovs.11-7370 21873683 PMC3183977

[185] Frenis K Helmstädter J Ruan Y Schramm E Kalinovic S Kröller-Schön S Ablation of lysozyme M-positive cells prevents aircraft noise-induced vascular damage without improving cerebral side effects. *Basic Res Cardiol.* (2021) 116:31. 10.1007/s00395-021-00869-5 33929610 PMC8087569

[186] Wang M Milic M Gericke A Mercieca K Liu H Ruan Y Chronic social defeat stress causes retinal vascular dysfunction. *Exp Eye Res.* (2021) 213:108853. 10.1016/j.exer.2021.108853 34800481

[187] Zadeh J Zhutdieva M Laspas P Yuksel C Musayeva A Pfeiffer N Apolipoprotein E deficiency causes endothelial dysfunction in the mouse retina. *Oxid Med Cell Longev.* (2019) 2019:5181429. 10.1155/2019/5181429 31781340 PMC6875001

[188] Gericke A Wolff I Musayeva A Zadeh J Manicam C Pfeiffer N Retinal arteriole reactivity in mice lacking the endothelial nitric oxide synthase (eNOS) gene. *Exp Eye Res.* (2019) 181:150–6. 10.1016/j.exer.2019.01.022 30716330

[189] Musayeva A Unkrig J Zhutdieva M Manicam C Ruan Y Laspas P Betulinic acid protects from ischemia-reperfusion injury in the mouse retina. *Cells.* (2021) 10:2440. 10.3390/cells10092440 34572088 PMC8469383

[190] Guevara-Torres A Joseph A Schallek J. Label free measurement of retinal blood cell flux, velocity, hematocrit and capillary width in the living mouse eye. *Biomed Opt Express.* (2016) 7:4228–49. 10.1364/boe.7.004228 27867728 PMC5102544

